# The obesity challenge in joint replacement: a multifaceted analysis of self-reported health status and exercise capacity using NHANES data: a population-based study

**DOI:** 10.1097/JS9.0000000000001287

**Published:** 2024-03-18

**Authors:** Xianzuo Zhang, Xianyue Shen, Jiaxiang Bai, Wanli Zang, Mo Chen, Abasi Maimaitiabula, Chen Zhu

**Affiliations:** a Department of Orthopedics, The First Affiliated Hospital of USTC, Division of Life Sciences and Medicine, University of Science and Technology of China, Hefei; b Postgraduate School, Harbin Sport University, Harbin, People’s Republic of China

**Keywords:** exercise capacity, joint replacement, obesity, self-reported health

## Abstract

**Background:**

Joint replacement is successful for end-stage oeteoarthritis, with obesity linked to elevated risk. But the impact of obesity on self-reported health and exercise capacity among joint replacement patients remains complex and requires investigation.

**Methods:**

This study utilizes data from the National Health and Nutrition Examination Survey (NHANES) to examine the relationship between obesity severity, demographic factors, medical comorbidities, and self-reported health status. The relationship between general health status and BMI was analyzed using multivariable regression, and further illustrated using a restricted cubic spline. Additionally, a bibliometric analysis and systematic review was done to frame the research within the broader context of existing knowledge and demographic specifics.

**Results:**

Analysis of NHANES data involving 327 joint replacement patients yielded intriguing insights. The difference in self-reported health between BMI groups did not achieve conventional statistical significance (*P*=0.06), and multivariable analysis showed that even severely obese patients did not exhibit significantly elevated risk of poor/fair self-reported health compared to normal weight subjects. Among severely obese individuals (BMI>40), 40.63% still rated their health positively. However, stratified analyses indicated that obesity correlated with negative health reports across sex, age, and education strata. Notably, physical functioning emerged as a robust predictor of self-reported health, with those reporting no walking difficulties having significantly lower odds of poor/fair health (Odds ratio=0.37, *P*=0.01).

**Conclusion:**

The study highlights the need for healthcare providers to consider individual physical abilities and comorbidities alongside obesity severity when discussing treatment options with joint replacement patients. It supports tailored interventions and informed shared decision-making. Future research could explore effective weight management strategies for obese individuals undergoing joint replacement.

## Introduction

Highlights

- Findings show that although obesity is often associated with poorer health outcomes, after joint arthroplasty surgery a significant portion (40.63%) of severely obese individuals still perceive their health positively. This contradicts conventional beliefs about the universally negative impact of severe obesity on health perception.
- The study highlights that exercise capacity diminishes with increasing obesity among joint replacement patients. It underscores the importance of assessing physical functioning when considering health status, challenging the notion that obesity is the sole determinant of health outcomes in these patients.
- Multivariable analysis indicates that even severely obese patients do not necessarily report poorer health than their normal weight counterparts. This suggests a more complex relationship between obesity and health perception than previously understood.
- Utilizing NHANES data, the study offers a representative analysis of the U.S. population, providing broader implications for public health and policy.

Joint replacement is a highly effective treatment for end-stage osteoarthritis. Globally, an estimated 38 million people have undergone total joint replacement surgery, with around 1.5 million of those procedures being performed in the United States (US) in 2020 alone. This figure is expected to reach 2.5 million by 2040^1^. Generally, patients who have undergone joint replacement surgery have a good life expectancy, with most of them experiencing significant improvements in pain and mobility. However, the success of the procedure depends on numerous factors, such as the patient’s age, overall health, and obesity level^2^.

Obesity is a global health issue and has been linked to several medical conditions, including osteoarthritis, which is a leading cause of joint replacement surgery. It has been shown that obesity increases the chances of total joint replacement surgery in adults^3^. However, those with severe obesity have a higher rate of postoperative complications following total joint arthroplasty (TJA), including a fourfold higher rate of infection, which can increase even further with more severe obesity^4–9^. These complications can be difficult for patients to manage and add billions of dollars of cost to the US annually^10^. As a result, severe obesity is increasingly being used as a relative contraindication to TJA despite the lack of alternative treatment options^11^. Therefore, surgeons may suggest that patients delay their elective joint arthroplasty procedure until they can reach a desired BMI in order to reduce their modifiable risk factors.

Self-reported health status of obese adults with joint replacement has been the focus of numerous studies, with the aim of understanding the impact of obesity on postsurgery outcomes and quality of life. Generally, the findings suggest that obese adults are at higher risk of postsurgery complications, with many studies finding that obese adults have lower self-reported physical function and quality of life compared to normal weight adults^12,13^. Additionally, obese adults tend to have higher rates of comorbidities and complications following surgery^5,8,9,11^. At the same time, there has been research that has found that obese adults can benefit from total joint replacement and experience similar physical activity and pain relief as those of normal weight. For instance, Li *et al*.^14^ found that obese participants experienced a significant amount of functional gain after joint replacement and experienced similar pain relief as those who were not obese. This suggests that with appropriate medical care and weight management strategies, obese adults can still benefit from total joint replacement.

This is still not known about the overall health and exercise capacity of obese joint replacement patients. Our hypothesis is that exercise capacity for obese joint replacement patients is likely to be reduced compared to those of normal weight. This is due to the higher risk of postsurgery complications and the associated physical limitations often experienced by obese patients. However, with appropriate medical care and weight management strategies, it is possible that obese patients could still benefit from increased exercise capacity and improved overall health. This would likely be dependent on the severity of the patient’s obesity, as well as the quality of their postoperative care. The purpose of this study is to investigate the self-reported health of obese adults with joint replacement, and to evaluate the impact of obesity on postsurgery outcomes and quality of life. In addition, the study aims to assess the exercise capacity of these patients, as well as to examine how medical care and weight management strategies can affect overall health and exercise capacity. This information could be used to develop strategies that would aid in enhancing shared decision-making and gaining a better understanding of patient satisfaction metrics.

## Materials and methods

The National Health and Nutrition Examination Survey (NHANES) is a survey that is conducted to obtain data from a representative sample of the noninstitutionalized, civilian US population. The survey is conducted every 2 years by the Centers for Disease Control and Prevention. This data is then used to produce national estimates and gain a better understanding of the health and nutrition of the US population. The survey uses a complex, multistage, probability sampling design. This means that counties are first randomly selected and then specific areas of the counties are randomly selected. Households within these areas and then individuals within these households are then randomly selected to participate. This ensures that the sample is representative of the US population and that each participant has an equal chance of being selected. Each participant is then assigned a sampling weight based on their likelihood of having been selected. This sampling weight is then used to produce national estimates based on the sample.

**Figure 1:**
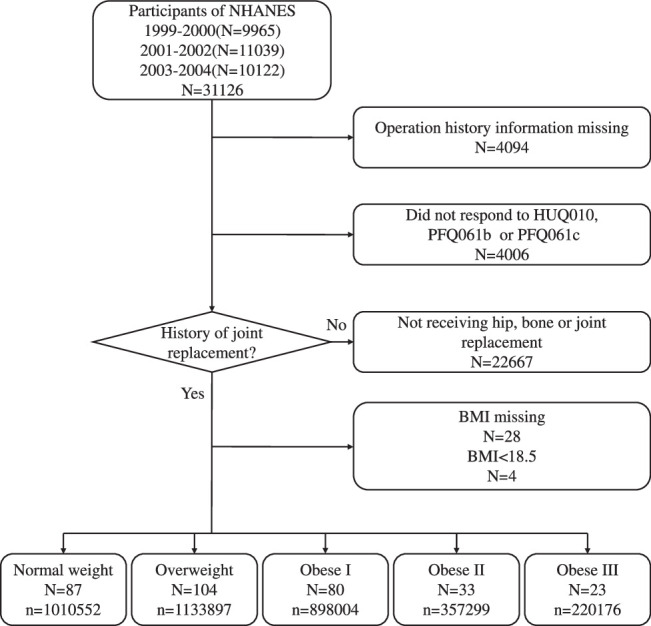
Flow chart of study population. (N represents the number of individuals surveyed; n represents the corresponding total population sample.

Using the NHANES data, we included participants in the analysis who were 18 years of age or older and had been identified as having joint replacement by a doctor or other health professional (OHQ148 asks, ‘Has a doctor ever told you that you have a hip, bone, or joint replacement?’). (Appendix S1, Supplemental Digital Content 1, http://links.lww.com/JS9/C183) To assess the study participants, data from three cycles spanning from 1 January 1999 to 31 December 2004 was used. NHANES conventionally measured the height and weight of the participants using a standardized digital scale (Fig. 1). The mobile examination center allowed for the collection of standardized information such as height and weight, as well as additional testing. In the conduct and reporting of our study, we have rigorously adhered to the Strengthening the Reporting of Cohort Studies in Surgery (STROCSS) (Supplemental Digital Content 2, http://links.lww.com/JS9/C184) criteria^15^.

The demographics, clinical, and functional characteristics of the included sample were described using both the absolute number of participants and the weighted number it represented in the US population, with percentages provided for the weighted results. Age was divided into 10-year groups for all analyses to ensure that its effect would not be assumed to be linear. Race and Hispanic ethnicity were combined into one variable, while education was divided into three categories: less than high school, high school degree, or college degree or more.

BMI was calculated by dividing a person’s body weight (in kilograms) by their height squared (in meters squared). It was then divided into five categories based on obesity thresholds: normal weight (BMI 18.5–25), overweight (BMI 25–29), stage I obesity (BMI 30–35), stage II obesity (BMI 35–39.9), and stage III obesity (BMI 40 and above). These thresholds were chosen as they have been proposed for limiting access to TJA and because they allowed for an adequate sample size and appropriate precision of association estimates (Appendix S2, Supplemental Digital Content 1, http://links.lww.com/JS9/C183). Other comorbidities examined were hypertension, diabetes mellitus (DM), hyperlipidemia, chronic obstructive pulmonary disease (COPD), asthma, asthma-COPD overlap syndrome (ACO), congestive heart failure, heart attack, chronic kidney disease (CKD), and alcohol associated liver disease. The questions used to assess these comorbidities are asked using multiple-choice questions, such as MCQ160e which asks, ‘Ever told you had heart attack?’. For complex disease diagnosis, a composite diagnostic approach was used, which combines medical history, blood pressure measurement, and antihypertensive medications (BPQ040A asks, ‘Because of your high blood pressure/hypertension, have you ever been told to take prescribed medicine?’).

Self-reported health was assessed in the NHANES with a Likert-like scale of five options: poor, fair, good, very good, and excellent. This was done through question HUQ010, which asks, ‘Would you say your health in general is?’. These responses were then divided into two categories: poor or fair health, and good, very good, or excellent health (Appendix S3, Supplemental Digital Content 1, http://links.lww.com/JS9/C183). In order to exhibit the exercise capacity of the participants, we also examined two functional outcomes: the ability to walk a quarter of a mile without help (PFQ061b asks, ‘By yourself and without using any special equipment, how much difficulty do you have walking a quarter of a mile?’) and the ability to climb 10 stairs without assistance (PFQ061c asks, ‘By yourself and without using any special equipment, how much difficulty do you have walking up 10 steps without resting?’).

To examine the relationship between demographic, clinical, and functional characteristics and self-reported health, frequencies and *χ*^2^ tests were used to assess univariable associations. Logistic regressions were then employed for multivariable associations, which included obesity severity alongside the strongest demographic, clinical, and functional predictors. Odds ratios and CIs were reported. To further analyze the results, sensitivity analyses were conducted on variables that were found to be significant in the multivariable regression model. Excluded demographic information in the flowchart is compared to the overall demographic information to exclude selection bias (Appendix S4, Supplemental Digital Content 1, http://links.lww.com/JS9/C183). Participants who had a poorer self-reported general health outcome were then compared to those with a better outcome, stratified by obesity severity. We also developed three nonlinear regression models to analyze the relationship between BMI (restricted cubic spline) and the self-reported health status. Ordinary Least Squares (OLS) regression was employed to ascertain the relationship between BMI and self-reported health status, and to formulate corresponding regression models. The OLS method endeavors to identify a linear equation (or a hyperplane in instances of multiple regression) that minimizes the sum of the squared deviations of the observed values from the predicted line. These deviations, quantified as the perpendicular distances from each observation to the fitted line, are termed residuals. The objective function governing the OLS estimation can be mathematically articulated as:


$${\sum_{i=1}^{n}\left(y_{i}-\left(a+b\cdot x_{i}\right)\right)}^{2}$$

where *y*_*i*​_ are the observations, *x*_*i*​_ are the explanatory variables, and *a* and *b* are the parameters of the model. Based on these assumptions, smoothed fitted curves were constructed to illustrate the relationship.

All statistical analyses were performed using the R language (R 4.1.3), with survey-specific commands selected, as well as cluster, strata, and weight information included. The weighting was adjusted to take into account the inclusion of multiple cycles. A precision estimate of 0.05 was chosen as the alpha threshold, and no changes were made to account for the examination of multiple predictors.

## Results

### Demographics and health conditions of obese participants with artificial joints

**Figure 2:**
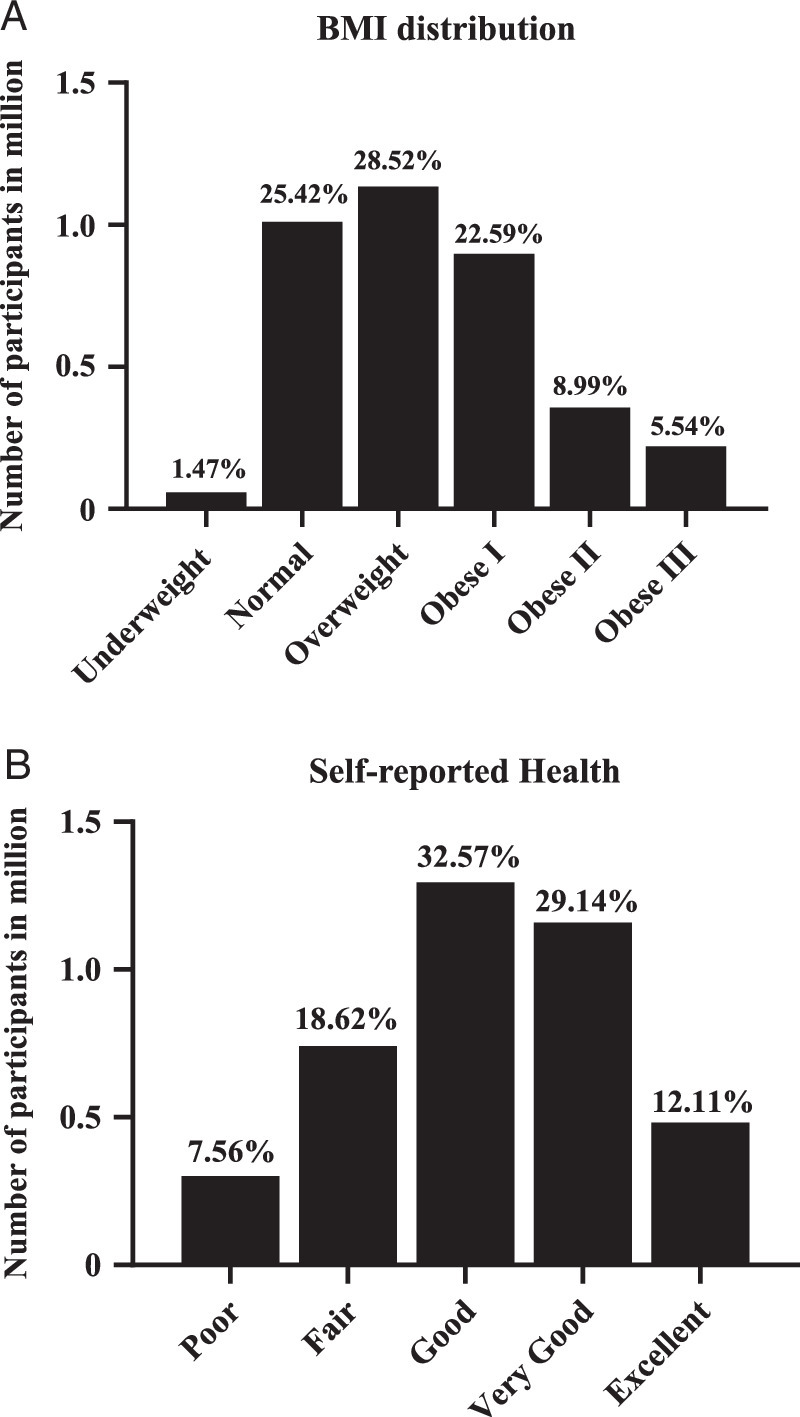
The BMI distribution of participants who underwent joint replacement (A) and self-reported health of the study population (B). The bars represent what would be expected in terms of a US population with these inclusion criteria and responses, while percentages are also included.

In total, 327 participants with artificial joints were included, representing a US population of 3 619 929. The number of men and women in the surveyed population was approximately equal, with an average age of 61.9±19.1 years. There was significant variation in demographic characteristics (Table 1). These patients often had other medical conditions and functional impairments. For example, 23.79% of these participants had hypertension and 7.43% had diabetes mellitus. Of the surveyed population, 25.42% were of a normal weight, 65.64% were overweight or obese, and 5.54% were severely obese (BMI>40) (Fig. 2A). All participants reported their general health status, with 74.47% of them reporting their health as good or better (Fig. 2B). Self-reported health status decreased as obesity increased, though 40.63% of participants with a BMI of 40 or higher still rated their health as good to excellent (Appendix S5, Supplemental Digital Content 1, http://links.lww.com/JS9/C183).

**Table 1:** Baseline characteristics of the study population.

	Normal BMI 18.5–25 *N*=1 010 552	Overweight BMI 25–29 N=1 133 898	Obese I BMI 30–35 N=898 004	Obese II BMI 35–39.9 N=357 299	Obese III BMI 40+N=220 176	*P*
Age						0.1
<40	313 548 (31.03)	280 421 (24.73)	83 572 (9.31)	91 510 (25.61)	55 107 (25.03)	
40–49	196 742 (19.47)	179 178 (15.80)	129 886 (14.46)	0 (0.00)	17 092 (7.76)	
50–59	146 091 (14.46)	122 930 (10.84)	229 492 (25.56)	82 451 (23.08)	57 551 (26.14)	
60–69	96 305 (9.53)	157 116 (13.86)	132 649 (14.77)	90 401 (25.30)	66 646 (30.27)	
70–79	105 415 (10.43)	269 960 (23.81)	215 441 (23.99)	78 067 (21.85)	23 780 (10.80)	
>79	152 451 (15.09)	124 293 (10.96)	106 964 (11.91)	14 870 (4.16)	0 (0.00)	
Sex						1
Female	487 611 (48.25)	579 532 (51.11)	390 702 (43.51)	192 295 (53.82)	126 080 (57.26)	
Male	522 940 (51.75)	554 365 (48.89)	507 302 (56.49)	165 004 (46.18)	94 096 (42.74)	
Race						0.3
Black, N-H	123 844 (12.26)	109 011 (9.61)	54 237 (6.04)	51 183 (14.32)	64 115 (29.12)	
White, N-H	830 027 (82.14)	859 528 (75.80)	761 655 (84.82)	253 012 (70.81)	146 674 (66.62)	
Hispanic	0 (0.00)	53 390 (4.71)	8098 (0.90)	0 (0.00)	0 (0.00)	
Mexican	27 295 (2.70)	37 069 (3.27)	22 462 (2.50)	36 455 (10.20)	9388 (4.26)	
Other	29 386 (2.91)	74 899 (6.61)	51 553 (5.74)	16 649 (4.66)	0 (0.00)	
Education						0.2
College degree or more	344 890 (34.13)	441 427 (38.93)	415 529 (46.27)	92 510 (26.27)	28 313 (12.86)	
High school degree	296 581 (29.35)	261 563 (23.07)	292 100 (32.53)	123 884 (35.18)	117 657 (53.44)	
Less than high school	369 081 (36.52)	430 907 (38.00)	190 375 (21.20)	135 760 (38.55)	74 206 (33.70)	
Income						0.1
Low (<less than 130%)	249 739 (27.44)	244 740 (25.59)	182 942 (22.26)	71 290 (22.92)	106 753 (50.10)	
Middle (131–338%)	379 992 (41.75)	337 782 (35.32)	298 331 (36.30)	199 179 (64.04)	101 789 (47.77)	
High (more than 338%)	280 453 (30.81)	373 928 (39.10)	340 533 (41.44)	40 559 (13.04)	4542 (2.13)	
Alcohol use						0.02
Never	154 789 (16.05)	207 594 (18.82)	33 671 (3.91)	48 142 (13.53)	70 618 (32.07)	
Former	234 025 (24.26)	194 834 (17.66)	205 881 (23.90)	165 834 (46.60)	117 616 (53.42)	
Mild	323 024 (33.49)	367 352 (33.31)	478 607 (55.56)	75 631 (21.25)	10 308 (4.68)	
Moderate	70 483 (7.31)	173 518 (15.73)	88 618 (10.29)	42 682 (11.99)	4542 (2.06)	
Heavy	182 277 (18.90)	159 654 (14.48)	54 634 (6.34)	23 564 (6.62)	17 092 (7.76)	
Smoke						0.3
Never	432 144 (44.44)	544 110 (48.24)	398 328 (44.36)	185 458 (51.91)	117 399 (53.32)	
Former	169 383 (17.42)	293 566 (26.03)	325 959 (36.30)	106 990 (29.94)	67 641 (30.72)	
Now	371 001 (38.15)	290 319 (25.74)	173 717 (19.34)	64 851 (18.15)	35 136 (15.96)	
Comorbidity						
Hypertension	350 310 (34.67)	515 252 (45.44)	544 270 (60.61)	276 886 (77.49)	201 424 (91.48)	<0.0001
Diabetes melilites	67 682 (6.94)	122 776 (11.03)	231 391 (25.77)	95 036 (26.74)	69 381 (31.51)	0.01
Hyperlipidemia	601 435 (59.52)	943 411 (83.20)	847 721 (94.40)	320 958 (89.83)	194 826 (88.49)	<0.0001
COPD	61 813 (6.36)	41 904 (3.71)	9595 (1.07)	14 349 (4.02)	39 056 (17.74)	0.1
Asthma	155 108 (15.35)	87 764 (7.74)	65 162 (7.26)	63 930 (17.89)	45 579 (20.70)	0.3
ACO	201 349 (20.70)	122 195 (10.83)	65 162 (7.26)	63 930 (17.89)	84 636 (38.44)	0.04
Heart attack	62 092 (6.43)	68 228 (6.05)	80 112 (8.99)	39 554 (11.07)	22 727 (10.32)	1
Congestive heart failure	6395 (0.67)	27 672 (2.45)	43 546 (4.85)	33 790 (9.46)	22 727 (10.32)	0.1
CKD	239 907 (24.75)	253 148 (23.86)	286 984 (32.15)	87 722 (24.55)	52 686 (23.93)	1
Alcohol associated liver disease	332 903 (32.94)	477 024 (42.07)	449 244 (50.03)	97 010 (27.15)	3069 (1.39)	0.03

### Exercise capacity in different BMI groups

Functional characteristics representing exercise capacity were compared in different BMI groups. Table 2 shows the results from this comparison, which show that for those with a BMI greater than 40 (which means they are considered obese), 5.61% were unable or had difficulty climbing 10 stairs – much higher than the 2.42% who had normal weight and did not have any difficulties in doing so. Similarly, 30.74% of those with an obesity-level BMI reported having some degree of difficulty walking a quarter mile while only 11.34% of individuals at normal weights experienced similar levels of struggle when performing this task. This information was used to assess the exercise capacity of the participants and to determine the level of difficulty they experienced in performing the activities.

**Table 2:** Functional characteristics representing exercise capacity of included participants.

	Normal BMI 18.5–25 *N*=1 010 552	Overweight BMI 25–29 *N*=1 133 898	Obese I BMI 30–35 *N*=898 004	Obese II BMI 35–39.9 *N*=357 299	Obese III BMI 40+*N*=220 176	*P*
Climb 10 stairs^a^						0.004
Unable or much difficulty	24 423 (2.42)	75 437 (6.65)	7799 (0.87)	85 487 (23.93)	12 352 (5.61)	
Some difficulty	114 582 (11.34)	127 827 (11.27)	96 114 (10.70)	20 465 (5.73)	67 688 (30.74)	
No difficulty	311 340 (30.81)	322 732 (28.46)	347 938 (38.75)	48 266 (13.51)	76 912 (34.93)	
Walk quarter mile^a^						0.004
Unable or much difficulty	55 950 (5.54)	98 017 (8.64)	35 327 (3.93)	72 589 (20.32)	49 167 (22.33)	
Some difficulty	88 921 (8.80)	72 431 (6.39)	112 555 (12.53)	40 084 (11.22)	87 163 (39.59)	
No difficulty	305 474 (30.23)	355 549 (31.36)	303 969 (33.85)	41 544 (11.63)	7092 (3.22)	

a Patients having to use an assist device were assumed to have much difficulty or be unable to do so.

### Factors influencing self-reported health status

Individual factors like sex, age, race, and education did not strongly correlate with self-reported health status. However, distinct univariate links emerged between various medical conditions such as hypertension, diabetes, COPD, heart attack, congestive heart failure, and CKD (Table 3). Regarding lifestyle habits, no direct connection between smoking and self-reported health was found, but there was a notable association between alcohol consumption and health perception. Moreover, having trouble in climbing 10 stairs and walking a quarter mile both showed individual associations with self-reported health.

**Table 3:** Univariable associations of participant characteristics with self-reported health.

	Self-reported health		
	Fair or poor	Good to excellent	
Characteristic	Weighted, *N* (%)	Weighted, *N* (%)	*P*
Sex			1
Female	447 453 (25)	1 328 768 (75)	
Male	476 677 (26)	1 367 031 (74)	
Age			0.1
<40	142 056 (17)	682 102 (83)	
40–49	98 578 (19)	424 320 (81)	
50–59	151 531 (24)	486 983 (76)	
60–69	178 217 (33)	364 900 (67)	
70–79	236 672 (34)	455 990 (66)	
>79	117 075 (29)	281 504 (71)	
Race/ethnicity			1
Black, N-H	115 996 (29)	286 393 (71)	
White, N-H	699 538 (25)	2 151 358 (75)	
Hispanic	17 330 (28)	44 159 (72)	
Mexican	39 305 (30)	93 363 (70)	
Other	51 961 (30)	120 526 (70)	
Education			0.1
College degree or more	202 467 (15)	1 120 202 (85)	
High school degree	326 595 (30)	765 190 (70)	
Less than high school	395 068 (33)	805 261 (67)	
Hypertension		0.002	
No	253 541 (15)	1 478 246 (85)	
yes	670 589 (36)	1 217 552 (64)	
DM			<0.001
DM	617 802 (22)	2 216 689 (78)	
IFG	23 902 (17)	117 266 (83)	
No	282 426 (48)	303 840 (52)	
Hyperlipidemia		1	
No	193 567 (27)	518 010 (73)	
Yes	730 563 (25)	2 177 789 (75)	
COPD			<0.0001
No	779 750 (23)	2 629 536 (77)	
Yes	143 149 (86)	23 569 (14)	
Asthma			0.2
No	772 145 (24)	2 430 241 (76)	
Yes	151 985 (36)	265 558 (64)	
Heart attack		<0.0001	
No	718 781 (22)	2 571 695 (78)	
Yes	197 568 (72)	75 145 (28)	
Congestive heart failure	<0.001		
No	808 118 (24)	2 620 056 (76)	
Yes	101 083 (75)	33 048 (25)	
CKD			0.004
No	527 810 (20)	2 052 278 (80)	
Yes	354 406 (39)	566 041 (61)	
Smoke			0.2
Former	325 403 (34)	638 137 (66)	
Never	383 762 (23)	1 293 676 (77)	
Now	213 734 (23)	721 291 (77)	
Alcohol use			0.002
Never	166 415 (32)	348 398 (68)	
Former	386 229 (42)	531 962 (58)	
Mild	165 255 (13)	1 089 667 (87)	
Moderate	37 507 (10)	342 337 (90)	
Heavy	116 713 (27)	320 508 (73)	
Climb 10 stairs			0.01
Unable or much difficulty	80 503 (39)	124 995 (61)	
Some difficulty	184 780 (43)	241 895 (57)	
No difficulty	198 104 (18)	909 084 (82)	
Walk quarter mile			<0.001
Unable or much difficulty	152 819 (49)	158 231 (51)	
Some difficulty	175 709 (44)	225 445 (56)	
No difficulty	134 859 (13)	878 770 (87)	

CHF, congestive heart failure; CKD, chronic kidney disease; COPD, chronic obstructive pulmonary disease; DM, diabetes mellites; IFG, impaired fasting glucose; N-H, non-Hispanic.

### Multivariable analysis of health status and predictors

**Figure 3:**
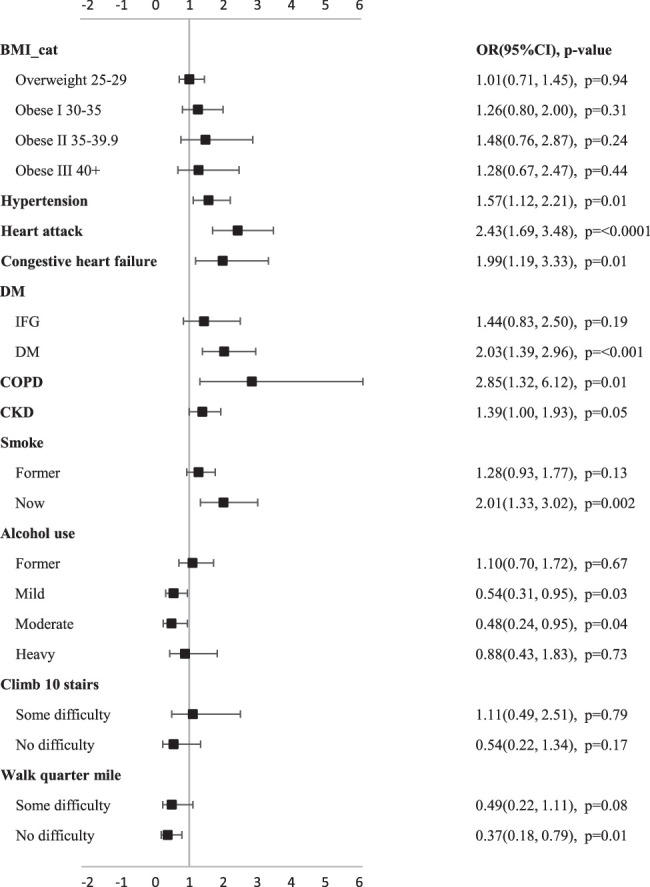
Forest plot of independent risk factors in multivariate Logistic regression analysis.

Multivariable analysis found that even severely obese patients with a BMI>40 did not have a significantly higher risk of self-reported poor or fair health compared to normal weight subjects (Fig. 3). The results of the multivariate analysis were consistent with the univariate analysis in terms of comorbidity and self-reported health. However, at the same time, patients who currently smoked were at an increased risk of poor or average self-reported health compared to nonsmokers (odds ratio=2.01 (95%CI:1.33, 3.02), *P*=0.002). The ability to walk a quarter mile emerged as a strong predictor of self-reported health, with those who had no difficulty walking being less likely to report poor or fair overall health than those who were unable to walk or had difficulty walking (odds ratio=0.37 (95%CI:0.18, 0.79), *P*=0.01).

### Sensitivity analysis and risk factors for poor health in specific groups

Further sensitivity analysis was conducted to stratify participants into different groups based on baseline characteristics, medical comorbidities, and exercise capacities. Obesity was found to be a risk factor for reporting worse general health status in both male and female groups. In females, overweight and obese I–III participants reported 1.477 (95% CI: 1.001–2.177, *P*=0.049), 3.264 (95% CI: 2.293–4.646, *P*<0.001), 3.468 (95% CI: 1.908–6.303, *P*<0.001), and 5.464 (95% CI: 2.953–10.109, *P*<0.001) times the risk of poor or fair general health, respectively, as compared to normal weight participants. Similar findings were observed in males. Overweight and obesity also increased the risk of negative health reports among non-Hispanic Whites, Blacks, and Mexicans. In the age-stratified subgroups, Class I–III obesity under 40 years of age increased the risk of reporting negative health conditions by 4.349 times (95% CI: 2.841–6.656), 6.185 times (95% CI: 3.241–11.805), and 5.738 times (95% CI: 2.673–12.316), respectively. Education levels, ability to climb 10 stairs and walk a quarter mile were also found to interact with obesity in the likelihood of reporting worse general health status (Table 4). The above results suggest potential association between BMI level and patient self-reported health status and confirm that this association was influenced by different levels of exercise capacity.

**Table 4:** Stratified analyses based on potential interactive variables.

	OR (95% CI)				
	Overweight	Obese I	Obese II	Obese III	
Characteristic	BMI 25–29	BMI 30–35	BMI 35–39.9	BMI 40+	*P*
Sex					0.28
Male	1.795 (1.230–2.621)	2.169 (1.370–3.436)	4.587 (2.506–8.396)	4.623 (2.197–9.728)	
Female	1.477 (1.001–2.177)	3.264 (2.293–4.646)	3.468 (1.908–6.303)	5.464 (2.953–10.109)	
Age					0.142
>79	0.982 (0.612–1.576)	2.060 (1.244–3.411)	1.225 (0.302–4.966)	8.163 (0.966–68.990)	
<40	1.513 (0.948–2.415)	4.349 (2.841–6.656)	6.185 (3.241–11.805)	5.738 (2.673–12.316)	
40–49	0.863 (0.425–1.753)	1.223 (0.536–2.793)	1.889 (0.628–5.682)	2.037 (0.471–8.819)	
50–59	0.865 (0.442–1.691)	0.958 (0.407–2.257)	1.811 (0.705–4.655)	1.814 (0.557–5.905)	
60–69	0.948 (0.570–1.576)	1.537 (0.816–2.896)	1.517 (0.568–4.052)	4.326 (1.629–11.485)	
70–79	0.769 (0.443–1.335)	0.992 (0.557–1.765)	1.816 (0.784–4.206)	1.485 (0.361–6.104)	
>79	0.982 (0.612–1.576)	2.060 (1.244–3.411)	1.225 (0.302–4.966)	8.163 (0.966–68.990)	
Race					0.378
White, n-h	1.557 (1.128–2.151)	2.630 (1.911–3.620)	4.184 (2.273–7.702)	4.733 (2.203–10.168)	
Mexican	2.102 (1.398–3.162)	2.114 (1.345–3.321)	2.219 (1.004–4.905)	4.586 (2.143–9.814)	
Black, n-h	1.615 (1.015–2.570)	2.349 (1.346–4.102)	3.386 (1.934–5.927)	5.446 (2.773–10.695)	
Other	0.688 (0.188–2.511)	2.216 (0.647–7.595)	3.335 (0.376–29.597)	17.032 (1.039–279.165)	
Hispanic	4.158 (1.006–17.177)	14.485 (3.079–68.138)	5.901 (0.490–71.073)	7.041 (0.500–99.203)	
Education					<0.001
College degree or more	0.781 (0.456–1.336)	1.839 (1.070–3.161)	2.738 (1.262–5.938)	1.695 (0.631–4.554)	
Less than high school	3.030 (2.254–4.072)	4.699 (3.264–6.765)	6.741 (3.621–12.549)	8.130 (4.752–13.910)	
High school degree	1.251 (0.825–1.896)	1.522 (0.926–2.501)	2.274 (1.252–4.130)	5.231 (2.184–12.526)	
Hypertension					<0.001
Yes	1.065 (0.750–1.512)	1.039 (0.721–1.496)	1.717 (0.981–3.003)	2.080 (1.198–3.614)	
No	1.206 (0.818–1.777)	3.432 (2.315–5.088)	3.133 (1.674–5.866)	3.186 (1.250–8.117)	
Heart attack					0.464
No	0.996 (0.757–1.310)	1.580 (1.126–2.217)	2.032 (1.247–3.312)	2.749 (1.544–4.895)	
Yes	0.850 (0.430–1.681)	1.117 (0.522–2.392)	3.183 (1.023–9.904)	6.829 (1.347–34.615)	
Congestive heart failure					0.546
No	0.977 (0.752–1.270)	1.535 (1.111–2.119)	2.071 (1.289–3.328)	2.570 (1.541–4.286)	
Yes	1.457 (0.528–4.018)	3.359 (1.282–8.798)	1.864 (0.476–7.305)	4.635 (0.603–35.617)	
DM					0.076
No	1.559 (1.206–2.015)	2.649 (1.888–3.717)	3.519 (2.092–5.917)	3.113 (1.690–5.734)	
IFG	1.358 (0.165–11.163)	1.513 (0.227–10.097)	1.250 (0.070–22.469)	13.717 (1.191–158.011)	
DM	0.704 (0.368–1.349)	0.665 (0.353–1.256)	1.207 (0.544–2.678)	1.322 (0.445–3.924)	
COPD					0.214
No	1.030 (0.788–1.347)	1.684 (1.225–2.315)	2.348 (1.423–3.872)	2.644 (1.578–4.431)	
Yes	0.966 (0.293–3.187)	0.993 (0.196–5.034)	0.933 (0.206–4.234)	32 987 429.685 (10 372 032.327–1.049139 e+08)	
CKD					0.728
No	1.222 (0.886–1.686)	1.750 (1.262–2.426)	2.702 (1.569–4.652)	3.166 (1.581–6.341)	
Yes	1.492 (0.916–2.428)	1.920 (1.278–2.883)	3.038 (1.562–5.910)	6.061 (2.738–13.416)	
Smoke					0.543
Never	1.132 (0.772–1.659)	1.799 (1.177–2.751)	3.346 (1.785–6.271)	4.497 (2.283–8.856)	
Former	0.814 (0.527–1.258)	1.453 (0.776–2.720)	1.975 (0.900–4.335)	1.937 (0.701–5.354)	
Now	1.137 (0.734–1.763)	1.710 (1.031–2.837)	1.341 (0.486–3.699)	2.335 (0.577–9.441)	
Alcohol use					0.225
Never	0.954 (0.498–1.826)	0.994 (0.461–2.141)	1.532 (0.513–4.576)	2.379 (0.789–7.169)	
Former	0.573 (0.324–1.012)	1.131 (0.576–2.220)	1.703 (0.756–3.835)	2.144 (0.850–5.405)	
Mild	1.572 (0.763–3.242)	1.956 (1.015–3.770)	2.230 (0.893–5.572)	1.648 (0.362–7.503)	
Moderate	0.602 (0.214–1.692)	1.614 (0.492–5.294)	5.762 (1.345–24.688)	2.536 (0.435–14.797)	
Heavy	2.594 (1.104–6.095)	2.908 (0.944–8.953)	2.837 (0.685–11.758)	3.077 (0.717–13.210)	
Climb 10 stairs					<0.001
Unable or much difficulty	0.739 (0.245–2.234)	1.475 (0.377–5.775)	0.809 (0.215–3.049)	3.972 (0.857–18.416)	
Some difficulty	0.453 (0.217–0.944)	0.605 (0.280–1.304)	1.464 (0.515–4.164)	0.829 (0.206–3.333)	
No difficulty	0.997 (0.641–1.550)	0.908 (0.493–1.672)	1.651 (0.724–3.765)	1.021 (0.203–5.141)	
Walk quarter mile					<0.001
Unable or much difficulty	0.273 (0.094–0.793)	0.388 (0.140–1.079)	0.483 (0.136–1.709)	1.145 (0.330–3.975)	
Some difficulty	0.427 (0.204–0.892)	0.615 (0.290–1.306)	1.536 (0.595–3.964)	0.927 (0.179–4.801)	
No difficulty	1.110 (0.718–1.716)	1.074 (0.580–1.990)	1.025 (0.245–4.295)	0.855 (0.263–2.782)	

### Exploring the relationship between BMI and health status

**Figure 4:**
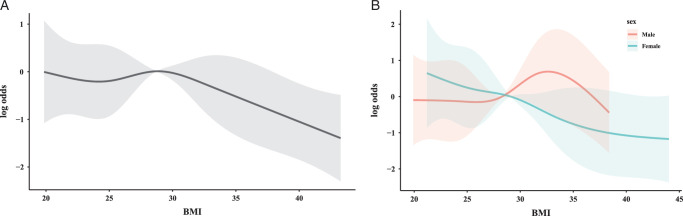
Correlation pattern between BMI and the likelihood of self-reported poor or fair health status for participants with different levels of exercise capacity. (A) Ability to walk quarter mile (B) Ability to climb 10 stairs.

In the investigation of the relationship between BMI and patient self-reported health status, an initial attempt was made to characterize this association through a linear model. However, the hypothesis test for general linear regression did not yield statistical significance (*P*>0.05), indicating that a linear model may not adequately capture the underlying relationship. Consequently, OLS regression was employed as a methodological approach to provide a more nuanced quantitative description of the association. The nonlinear nature of the relationship was further explored and visualized using several smooth-fitting curves, with BMI being modeled as a restricted cubic spline. The findings revealed that subjects were less inclined to report good or excellent health status when their BMI exceeded 29 (Fig. 4A), with variations observed between genders. Specifically, male subjects were more likely to report favorable self-status when their BMI was greater than 28, a likelihood that increased until it reached a peak at 34, and then gradually declined, reverting to the reference value at 37. Conversely, among female subjects, the probability of reporting a positive self-state was found to diminish progressively as BMI increased (Fig. 4B).

### Additional analysis

A bibliometric analysis was undertaken to assess the breadth of literature on the influence of obesity on the outcomes of arthroplasty surgery, as delineated in Appendices S6.1 and S6.2. Results indicated an upward trend in the volume of publications and their corresponding citations over time, as documented in Appendix S6.3 (Supplemental Digital Content 1, http://links.lww.com/JS9/C183). The journals with the highest number of publications in this domain were identified as *Clinical Orthopaedics and Related Research*, *the Journal of Arthroplasty*, and *the Journal of Bone and Joint Surgery-American Volume* (Appendix S6.4, Supplemental Digital Content 1, http://links.lww.com/JS9/C183). Scholars from the United States have contributed the most significant number of influential publications in this area (Appendix S6.5, Supplemental Digital Content 1, http://links.lww.com/JS9/C183), with the Hospital for Special Surgery emerging as the leading institution (Appendix S6.6, Supplemental Digital Content 1, http://links.lww.com/JS9/C183). Additionally, an examination of the articles’ publication data by country and area, as well as the collaborative networks between states, institutions, and authors, was conducted (Appendices S6.7-9), providing insight into the global research landscape and the extent of collaborative efforts within this field.

The systematic review yield 39 studies examining the relationship between obesity and outcomes of joint arthroplasties (Appendix S7.1 and 7.2, Supplemental Digital Content 1, http://links.lww.com/JS9/C183). A diverse range of patient populations and surgical techniques were analyzed. It was highlighted that obesity is associated with longer operative times, higher complication rates, and increased risk of readmission and revision surgery. Notably, however, some studies indicated that obese patients could still experience significant improvements postsurgery, comparable to nonobese counterparts. The timing of bariatric surgery prior to joint arthroplasty did not significantly alter complication profiles, and weight loss prior to surgery was associated with postoperative weight gain, suggesting a need to re-evaluate current preoperative weight loss recommendations. Collectively, these findings emphasize the complex interplay between obesity and arthroplasty outcomes and underscore the importance of individualized patient care.

## Discussion

Given the increasing prevalence of obesity in the US, it is likely that obese patients will continue to seek arthroplasty in the future. It is important to understand how to best optimize this challenging clinical interaction. This study looked at the self-reported health of a representative sample of US adults who had arthroplasty and identified as overweight or obese by BMI. The results showed that obese participants were more likely to report poorer general health status and demonstrate limited exercise capacity, yet over 40% of obese patients still described themselves as being in good to excellent health. When consulting with obese patients, clinicians should inform them of the risks associated with obesity and the potential benefits of undergoing surgery, and collaborate with them to decide if an elective TJA surgery should be delayed.

Patients with severe arthropathy, such as osteoarthritis and osteonecrosis of femoral head, who are severely obese are usually referred for TJA because other nonsurgical treatments have failed to provide sufficient symptom relief. Weight loss is a very effective way of reducing these symptoms, improving gait mechanics, and decreasing the likelihood of complications after TJA^7,16–18^. However, weight loss can be difficult, and many patients may not have the resources to manage it. Therefore, in addition to educating patients on the health impacts of obesity, more research and public health initiatives are needed to help pair patients with severe obesity and a desire to lose weight with programs that can assist them. Doing so may be the best opportunity to connect them with such a plan. Moreover, additional research is necessary to determine the extent to which weight loss can actually modify the risks associated with TJA^19^.

It was found that among American adults with osteoarthritis and a BMI over 45, more than half still reported their health to be good or better^20^. However, among severely obese joint replacement patients in this study, the relevant percentage dropped to 40.63%. The proportion of people who reported their health as ‘Very Good’ or ‘Excellent’ fell from 39.22% and 11.95% to 9.43% and 3.47%, respectively. Many studies have confirmed the association between severe obesity and poor health outcomes, including not only complications following TJA^4–8^, but also all-cause mortality^21,22^. For orthopedic surgeons or health providers, this may be common knowledge; however, for patients, this may come as a surprise. It may be helpful for surgeons to not assume that patients already have this understanding when they enter these encounters. Our results confirm the hypothesis that exercise capacity for obese joint replacement patients is likely to be reduced compared to those of normal weight. This aligns with previous research, such as the work by Li *et al*.^14^. The increased risk of postoperative complications in obese patients is well-documented, but our study further elucidates the impact on self-reported health and exercise capacity.

The associations between obesity severity, patient characteristics, and self-reported health present a multifaceted picture. Demographic factors such as age and economic income are known to have an effect on health. Chronic diseases such as diabetes and hypertension are even more important when considering health status. Additionally, lifestyle factors such as smoking and alcohol consumption can also impact overall health status. In this study, data from the NHANES database was used to adjust for mediators and confounders that could influence the self-reported health status of joint replacement patients. Our study reveals that even among severely obese individuals, a significant proportion perceive their health as good or better, with physical functioning emerging as a strong predictor. Unlike some previous research that found demographic factors like age and sex to be significant, our analysis emphasizes the role of comorbidities such as hypertension and diabetes. These findings challenge conventional assumptions and underscore the complexity of self-perception in health among obese individuals. The insights call for personalized interventions and counseling that consider not only the degree of obesity but also individual physical abilities and chronic conditions. This nuanced understanding can inform targeted strategies to improve overall health outcomes and contribute to more effective shared decision-making in clinical practice.

While medical comorbidities such as cardiovascular disease and stroke were associated with decreased self-reported health, measures of physical functioning were still a strong predictor. Physical inactivity leads to a decline in functional capacity, including decreases in cardiorespiratory fitness, strength, balance, and flexibility, all of which are associated with decreased self-reported health. These physical functioning limitations are particularly pronounced in obese individuals, where severity correlates with a higher risk of complications and lower quality of life.

Given the complex link between obesity severity, patient characteristics, and self-reported health, the lack of insight into overall health status is an important discovery and can be a potential obstacle in communication. This further emphasizes the importance of helping patients understand the correlation between severe obesity and overall health. It is likely that many patients assume that undergoing TJA and improved function will result in better general health. Although medical comorbidities, arthropathy, and physical function are all linked, these relationships can be complex. For example, patients should not assume that they will lose weight after TJA, as this is a common misconception^16,23–25^. Further research is still needed to figure out the best way to communicate this intricate information.

These results point to a potential issue associated with using patient satisfaction surveys or customer rating systems to gauge the quality of medical care. In some healthcare systems, patients who are severely obese may not even have access to referral to an orthopedic surgeon and evaluation for TJA. It is unknown how these referral patterns affect access to weight loss programs and how interested patients are in them. For those who do show up for surgical evaluation, they may be discouraged or unsatisfied to learn that their weight is a relative contraindication to TJA, especially if they have been told TJA is their last remaining treatment option and have not received counseling about their weight beforehand. Quality care assessments meant to evaluate physician performance may be biased against practices that have this kind of high-risk patients. Previous research is limited but has indicated that patients’ perception of care may be more connected to interpersonal communication than quality^26,27^. In a clinical setting, being a repeat patient appears to be the strongest known predictor of satisfaction. Studies on TJA have only established a weak connection between patient satisfaction with care at the time of TJA and outcomes at 2 years postoperatively and that initial satisfaction is largely driven by pain^28,29^.

The results of this study should be interpreted in light of several limitations. While the NHANES database offers valuable insights into the health status of the U.S. population, we acknowledge certain limitations in scaling data from a sample of over 300 individuals to represent over 3 million. This extrapolation, although methodologically sound and common in epidemiological studies, might not capture subtle nuances present in the larger population. We employed robust statistical methods for data weighting and stratification to mitigate these limitations. However, it is important for readers to consider these aspects when interpreting the study’s findings. Future research with larger, direct sample sizes could further validate and expand upon our results. Using NHANES data, it is impossible to determine which specific joints were replaced, such as hip or knee replacements. Furthermore, the state of the artificial joint at the time of the survey was also unknown. In addition, by limiting the sample to those with functional limitations, it is likely that patients with more severe disease were selected; however, this cannot be verified. It was also not possible to know how long these patients had their arthroplasty surgery. Despite these limitations, it is unclear how they would impact the study’s results. For example, patients who have had hip and knee replacements and have the same functional limitations may have similar views of their self-reported health. In contrast, those with radiographically progressed prosthetic wear who have the same functional limitations may also not have dissimilar views of their self-reported health. However, a key strength of this study was the quality of the NHANES data. It uses a population-based cluster random selection method to collect data from a sample representative of the entire US population. Data is collected with rigorous quality control and assurance measures, such as height and weight measurements collected by trained staff.

## Conclusion

In obese adults with joint replacement, over 40% reported positive health outcomes, suggesting that comprehensive care and lifestyle changes can offset obesity-related health risks. This highlights the importance of holistic patient counseling that includes weight management and exercise for improved postoperative satisfaction and health.

## Ethical approval

Data for this study were obtained from the NHANES database. Related Ethical approval could be found at *NHANES Data Release and Access Policy*.

## Consent

NIH obtained written consent from subjects for the collection and utility data.

## Sources of fundings

This work was supported by the National Natural Science Foundation of China (Grant No. 82202672), the Key Research and Development Program of Anhui Province (No. 2022e07020017), China Postdoctoral Science Foundation Grant (2022M723049), the Natural Science Foundation of Anhui Province(2108085QH319), Anhui Provincial Research Preparation Plan (2022AH040074) and the Fundamental Research Funds for the Central Universities (WK9110000173).

## Author contribution

Z.X., S.X., and B.J.: conceived the idea for this study; Z.X., S.X., A.M., and C.M.: carried out the data extraction; Z.X., Z.W., and S.X.: statistical analysis; Z.X. and B.J.: image plotting; Z.X., Z.W., A.M., and C.M.: drafted the initial manuscript, while all co-authors provided critical feedback and revisions to the final version; Z.X. and Z.C.: provided financial support; Z.C.: provided administrative assistance.

## Conflicts of interest disclosure

None of the authors have any conflicts to report.

## Research registration unique identifying number (UIN)

This study employs a data mining approach using the National Health and Nutrition Examination Survey (NHANES) database. NHANES is a program of studies designed to assess the health and nutritional status of adults and children in the United States, and it is already a registered and publicly accessible database. The data in NHANES have been collected with adherence to the registry requirements set forth for studies involving human participants. As our study does not involve the direct recruitment of human subjects but instead analyzes pre-existing, de-identified data from this comprehensive registry, the requirement for an additional Unique Identifying Number (UIN) registration for this submission can be considered waived. Our research conforms to the principles outlined in the World Medical Association's Declaration of Helsinki, specifically article 35, as the NHANES database itself is registered and complies with the necessary ethical and methodological standards for human subject research.

## Guarantor

Zhang Xianzuo and Zhu Chen.

## Data availability statement

*Availability of data and material:* The data supporting the findings of this study are available within the article and its supplementary materials. Additional datasets generated during and/or analyzed during the current study are available are available from the corresponding author on reasonable request.

*Code availability (software application or custom code):* All analyses were conducted using R (version [4.1.3]). Specific scripts and commands used in the analysis are available upon request.

*Informed consent:* Informed consent was obtained from all individual participants included in the study.

## Provenance and peer review

Not commissioned, externally peer-reviewed.

## Supplementary Material
