# Supplementary material for: The obesity challenge in joint replacement: a multifaceted analysis of self-reported health status and exercise capacity using NHANES data: a population-based study
Source: Int J Surg. 2024 Mar 18;110(6):3212–22. doi: 10.1097/JS9.0000000000001287 (PMC11175787; doi:10.1097/JS9.0000000000001287)
Supplement: Supplementary file 1 [file js9-110-3212-s001.docx]

Supplementary Materials for

**The Obesity Challenge in Joint Replacement:**

**A Multifaceted Analysis of Self-reported Health Status and Exercise Capacity Using NHANES Data - A Population-Based Study**

***This document includes:***

**Appendix S1.** Definitions of joint replacement in this population-based study.

**Appendix S2.** Definitions of obesity based on BMI

**Appendix S3.** Definition of self-reported health status according to the NHANES questionnaire.

**Appendix S4.** Demographic comparison between the excluded groups in the flow chart and the population as a whole

**Appendix S5.** Self-reported health in patients with different BMI.

**Appendix S6.** Systemic search and bibliometric study of obesity influence on arthroplasty surgical outcomes.

**Appendix S6.1** Searched databases

**Appendix S6.2** Search strategy

**Appendix S6.3** Annual publications and citations base on WOS^TM^.

**Appendix S6.4** The top 10 most productive journals

**Appendix S6.5** The top 10 most productive countries

**Appendix S6.6** The most productive institutions

**Appendix S6.7** Publication of articles counted by countries and areas.

**Appendix S6.8** Cooperation between States, institutions, and authors

**Appendix S6.9**

**Appendix S7.** Systemic review of influence of obesity on surgical outcomes of joint replacement.

**Appendix S7.1** PRISMA flow chart of study identification, screening, and selection for the obesity and joint replacement surgical outcomes

**Appendix S7.2** Comprehensive review chart of systemically selected study populations, findings, and recommendations.

**Appendix S1.** Definitions of joint replacement in this population-based study.

OHQ148 - Have a hip, bone, or joint replacement?

Variable Name: OHQ148

SAS Label: Have a hip, bone, or joint replacement?

English Text: Q12. Has a doctor ever told you that you have a hip, bone, or joint replacement?

Target: Both males and females 12 YEARS - 150 YEARS

Year 1999-2000

| Code or Value | Value Description | Count | Cumulative | Skip to Item |
| --- | --- | --- | --- | --- |
| 1 | Yes | 159 | 159 |  |
| 2 | No | 5588 | 5747 |  |
| 7 | Refused | 0 | 5747 |  |
| 9 | Don't know | 0 | 5747 |  |
| . | Missing | 2840 | 8587 |  |

Year 2001-2002

| Code or Value | Value Description | Count | Cumulative | Skip to Item |
| --- | --- | --- | --- | --- |
| 1 | Yes | 113 | 113 |  |
| 2 | No | 5980 | 6093 |  |
| 7 | Refused | 0 | 6093 |  |
| 9 | Don't know | 0 | 6093 |  |
| . | Missing | 3505 | 9598 |  |

Year 2003-2004

| Code or Value | Value Description | Count | Cumulative | Skip to Item |
| --- | --- | --- | --- | --- |
| 1 | Yes | 87 | 87 |  |
| 2 | No | 5481 | 5568 |  |
| 7 | Refused | 0 | 5568 |  |
| 9 | Don't know | 0 | 5568 |  |
| . | Missing | 3279 | 8847 |  |

**Appendix S2.** Definitions of obesity based on BMI

Body Mass Index (BMI) is a universally recognized metric for categorizing body weight relative to height. It is computed by dividing a person's weight in kilograms by their height in meters squared. This simple formula has become a standard tool in assessing obesity, which is critical in various medical and clinical research fields, including studies related to Total Joint Arthroplasty (TJA).

| BMI Range | Category | Description |
| --- | --- | --- |
| 18.5 - 25 | Normal Weight | Individuals within this range are considered to have an ideal body weight relative to their height. |
| 25 - 29 | Overweight | Represents individuals who are above the ideal weight for their height but not classified as obese. |
| 30 - 35 | Stage I Obesity | The initial stage of obesity, indicating a significant divergence from the ideal weight range. |
| 35 - 39.9 | Stage II Obesity | A more severe level of obesity, associated with greater health risks. |
| 40 and above | Stage III Obesity | The most severe category, indicating extremely high levels of obesity and often associated with serious health concerns. |

The rationale behind these specific thresholds revolves around their relevance in limiting access to Total Joint Arthroplasty (TJA). These ranges not only provide a structured approach to classifying obesity but also ensure an adequate sample size and precision in estimating associations in clinical studies. By standardizing these categories, researchers and healthcare providers can make more informed decisions regarding treatment plans and understand the potential impacts of obesity on surgical outcomes and overall health.

**Appendix S3.** Definition of self-reported health status according to the NHANES questionnaire.

HUQ010 - General health condition

Variable Name: HUQ010

SAS Label: General health condition

English Text: {First/Next} I have some general questions about {your/SP's} health. Would you say {your/SP's} health in general is . . .

English Instructions: CAPI INSTRUCTION: DISPLAY "FIRST" IF SP AGE IS >= 16 YEARS.

Target: Both males and females 0 YEARS - 120 YEARS

**Year 1999-2000**

| Code or Value | Value Description | Count | Cumulative | Skip to Item |
| --- | --- | --- | --- | --- |
| 1 | Excellent, | 3002 | 3002 |  |
| 2 | Very good, | 2464 | 5466 |  |
| 3 | Good, | 2867 | 8333 |  |
| 4 | Fair, or | 1306 | 9639 |  |
| 5 | Poor? | 316 | 9955 |  |
| 7 | Refused | 0 | 9955 |  |
| 9 | Don't know | 5 | 9960 |  |
| . | Missing | 5 | 9965 |  |

**Year 2001-2002**

| Code or Value | Value Description | Count | Cumulative | Skip to Item |
| --- | --- | --- | --- | --- |
| 1 | Excellent, | 3728 | 3728 |  |
| 2 | Very good, | 2855 | 6583 |  |
| 3 | Good, | 2902 | 9485 |  |
| 4 | Fair, or | 1259 | 10744 |  |
| 5 | Poor? | 290 | 11034 |  |
| 7 | Refused | 3 | 11037 |  |
| 9 | Don't know | 2 | 11039 |  |
| . | Missing | 0 | 11039 |  |

**Year 2003-2004**

| Code or Value | Value Description | Count | Cumulative | Skip to Item |
| --- | --- | --- | --- | --- |
| 1 | Excellent, | 3273 | 3273 |  |
| 2 | Very good, | 2535 | 5808 |  |
| 3 | Good, | 2788 | 8596 |  |
| 4 | Fair, or | 1225 | 9821 |  |
| 5 | Poor? | 297 | 10118 |  |
| 7 | Refused | 3 | 10121 |  |
| 9 | Don't know | 1 | 10122 |  |
| . | Missing | 0 | 10122 |  |

**Appendix S4.** Demographic comparison between the excluded groups in the flow chart and the population as a whole
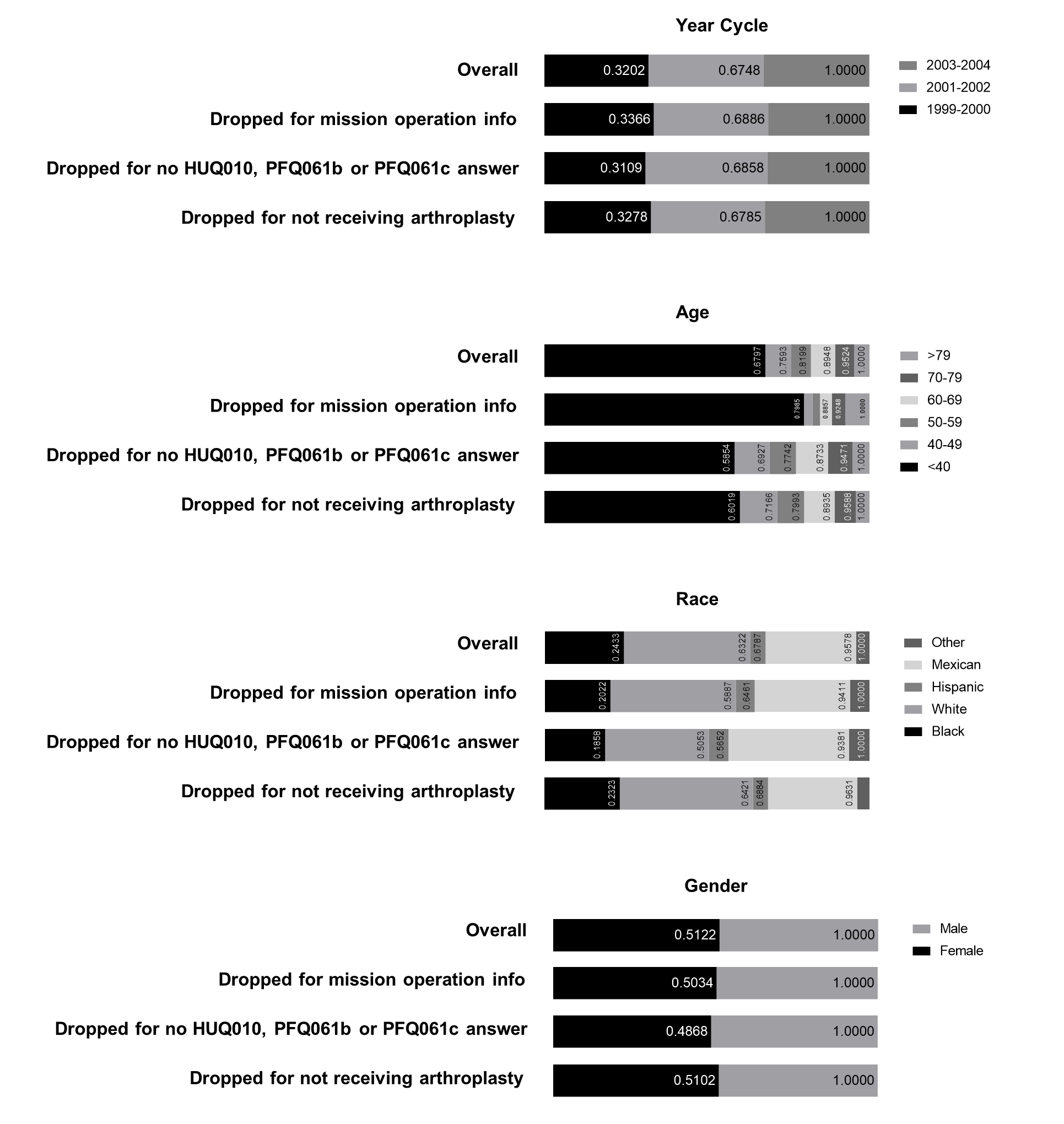


Data exclusion due did not affect the inclusion of demographic information bias between excluded subjects and the total NHANES study population

**Appendix S5.** Self-reported health in patients with different BMI.

| Obesity Class | Self-Reported Health | | | | | P-value |
| --- | --- | --- | --- | --- | --- | --- |
|  | Poor | Fair | Good | Very Good | Excellent |  |
|  | Weighted, N (%) | Weighted, N (%) | Weighted, N (%) | Weighted, N (%) | Weighted, N (%) |  |
| BMI |  |  |  |  |  | 0.06 |
| Normal 18.5-25 | 65218(6.45) | 165326(16.36) | 262935(26.02) | 396320(39.22) | 120754(11.95) |  |
| Overweight 25-29 | 62554(5.52) | 152978(13.49) | 333615(29.42) | 363932(32.10) | 220818(19.47) |  |
| Obese I 30-35 | 39990(4.45) | 193636(21.56) | 367352(40.91) | 229579(25.57) | 67447(7.51) |  |
| Obese II 35-39.9 | 22086(6.18) | 91625(25.64) | 159489(44.64) | 72619(20.32) | 11480(3.21) |  |
| Obese III 40+ | 24243(11.01) | 106474(48.36) | 61058(27.73) | 20767(9.43) | 7634(3.47) |  |

**Appendix S6.** Systemic search and bibliometric study of obesity influence on arthroplasty surgical outcomes.

We searched the studies for obesity and joint replacement surgical outcomes through the Web of Science™ on Wed Jan 10 2024. The searched databases were summarized in Appendix S6.1. Search strategy was shown in Appendix S6.2.

**Appendix** S6.1 Searched databases

| Database | Coverage Period |
| --- | --- |
| Web of Science (WoS) | 1900 to 2024 |
| Chinese Science Citation Database (CSCD) | 1989 to 2024 |
| Derwent Innovations Index (DIIDW) | 1966 to 2024 |
| INSPEC | 1969 to 2024 |
| Korea Journal Database (KJD) | 1980 to 2024 |
| MEDLINE | 1950 to 2024 |
| ProQuest Publicly Available Content Database (PPRN) | 1991 to 2024 |
| ProQuest Dissertations & Theses Global (PQDT) | 1637 to 2024 |
| Scientific Electronic Library Online (SCIELO) | 2002 to 2024 |

**Appendix** S6.2 Search strategy

| # | Search Query | Results |
| --- | --- | --- |
| 1 | TI=(obesity) | 186925 |
| 2 | TS=(High BMI) | 167019 |
| 3 | TS=(Overweight) | 185806 |
| 4 | #3 OR #2 OR #1 | 449440 |
| 5 | TS=(Arthroplasty) | 151911 |
| 6 | TS=(Total Knee Replacement) | 47603 |
| 7 | TS=(Total Hip Replacement) | 58895 |
| 8 | TS=(Joint Reconstruction) | 79917 |
| 9 | TS=(Orthopedic Surgery) | 78293 |
| 10 | TS=(Prosthesis or Prosthetic Joint) | 491749 |
| 11 | TS=(Artificial Joint) | 91377 |
| 12 | #11 OR #10 OR #9 OR #8 OR #7 OR #6 OR #5 | 795700 |
| 13 | TS=(Surgical Outcomes) | 648410 |
| 14 | TS=(Patient Satisfaction) | 237452 |
| 15 | TS=(Quality of Life) | 1227381 |
| 16 | TS=(General Health) | 3362105 |
| 17 | #16 OR #15 OR #14 OR #13 | 5170414 |
| 18 | (TI=(guideline)) OR AB=(guideline) | 872214 |
| 19 | (TI=(guidance)) OR AB=(guidance) | 601088 |
| 20 | (TI=(position paper)) OR AB=(position paper) | 634550 |
| 21 | (TI=(position stand)) OR AB=(position stand) | 162480 |
| 22 | (TI=(statement)) OR AB=(statement) | 320494 |
| 23 | (TI=(recommendation)) OR AB=(recommendation) | 925802 |
| 24 | (TI=(consensus)) OR AB=(consensus) | 398427 |
| 25 | (TI=(practice parameter)) OR AB=(practice parameter) | 123969 |
| 26 | (TI=(standards)) OR AB=(standards) | 4477254 |
| 27 | (TI=(management)) OR AB=(management) | 5134188 |
| 28 | #27 OR #26 OR #25 OR #24 OR #23 OR #22 OR #21 OR #20 OR #19 OR #18 | 12380786 |
| 29 | #4 AND #17 AND #12 AND #28 | 372 |

**Appendix S6.3** Annual publications and citations base on WOS^TM^.

**
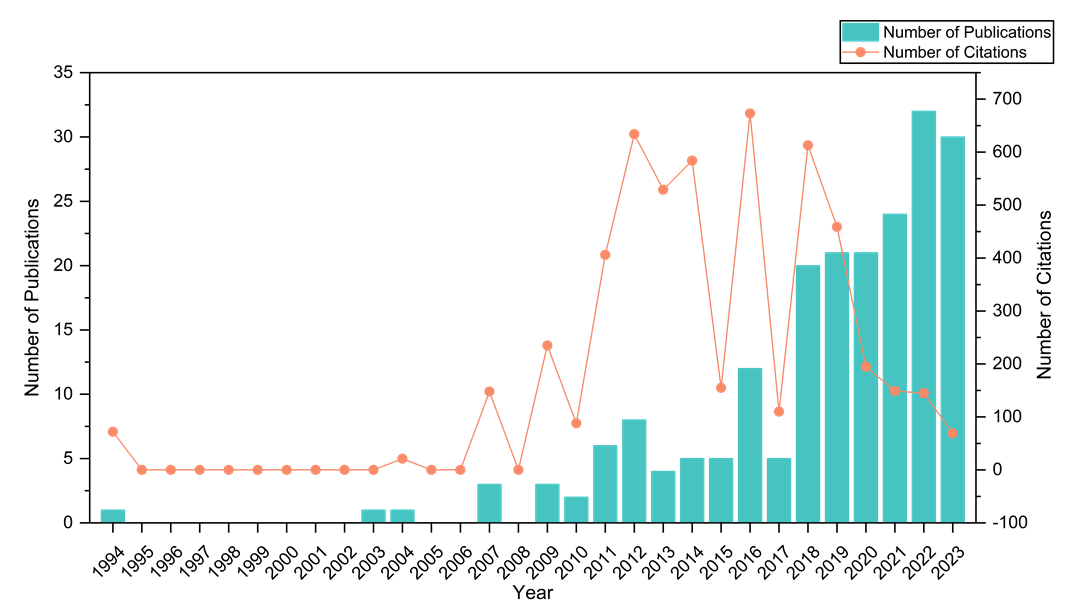
**

| **Appendix S6.4** The top 10 most productive journals | | | | |
| --- | --- | --- | --- | --- |
| Journal | Publications | Total citations | Average citation | Impact factor |
| Clinical Orthopaedics and Related Research | 17 | 470 | 27.65 | 4.3 |
| Journal of Arthroplasty | 14 | 335 | 23.93 | 3.5 |
| Journal of Bone and Joint Surgery-American Volume | 11 | 1234 | 112.18 | 5.3 |
| Knee Surgery Sports Traumatology Arthroscopy | 8 | 94 | 11.75 | 3.8 |
| BMC Musculoskeletal Disorders | 7 | 122 | 17.43 | 2.3 |
| Osteoarthritis and Cartilage | 6 | 376 | 62.67 | 7 |
| Hip International | 5 | 30 | 6.00 | 1.5 |
| European Journal of Orthopaedic Surgery and Traumatology | 4 | 36 | 9.00 | 1.7 |
| Journal of Orthopaedics | 4 | 12 | 3.00 | 1.5 |
| Journal of Clinical Medicine | 4 | 3 | 0.75 | 3.9 |

| **Appendix S6.5** The top 10 most productive countries | | | | |
| --- | --- | --- | --- | --- |
| Country | Articles | Total citations | Average citations | Percentage |
| United States | 98 | 2679 | 27.34 | 48.04 |
| Germany | 20 | 211 | 10.55 | 9.80 |
| United Kingdom | 19 | 759 | 39.95 | 9.31 |
| Italy | 15 | 177 | 11.80 | 7.35 |
| Canada | 13 | 347 | 26.69 | 6.37 |
| Australia | 12 | 478 | 39.83 | 5.88 |
| Switzerland | 9 | 115 | 12.78 | 4.41 |
| Netherlands | 8 | 591 | 73.88 | 3.92 |
| China | 8 | 39 | 4.88 | 3.92 |
| France | 7 | 52 | 7.43 | 3.43 |

| **Appendix S6.6** The most productive institutions | | | | |
| --- | --- | --- | --- | --- |
| Institution | Country | Articles | Total citations | Average citations |
| Hospital for Special Surgery | United States | 6 | 251 | 41.83 |
| The University of Sydney | Australia | 5 | 169 | 33.80 |
| Massachusetts General Hospital | United States | 5 | 135 | 27.00 |
| Stanford University | United States | 5 | 94 | 18.80 |
| Harvard Medical School | United States | 5 | 56 | 11.20 |
| Mayo Clinic | United States | 5 | 56 | 11.20 |
| Keele University | United Kingdom | 4 | 62 | 15.50 |
| Northwestern University | United States | 4 | 61 | 15.25 |
| The University of Maryland | United States | 4 | 20 | 5.00 |

**Appendix S6.7** Publication of articles counted by countries and areas.


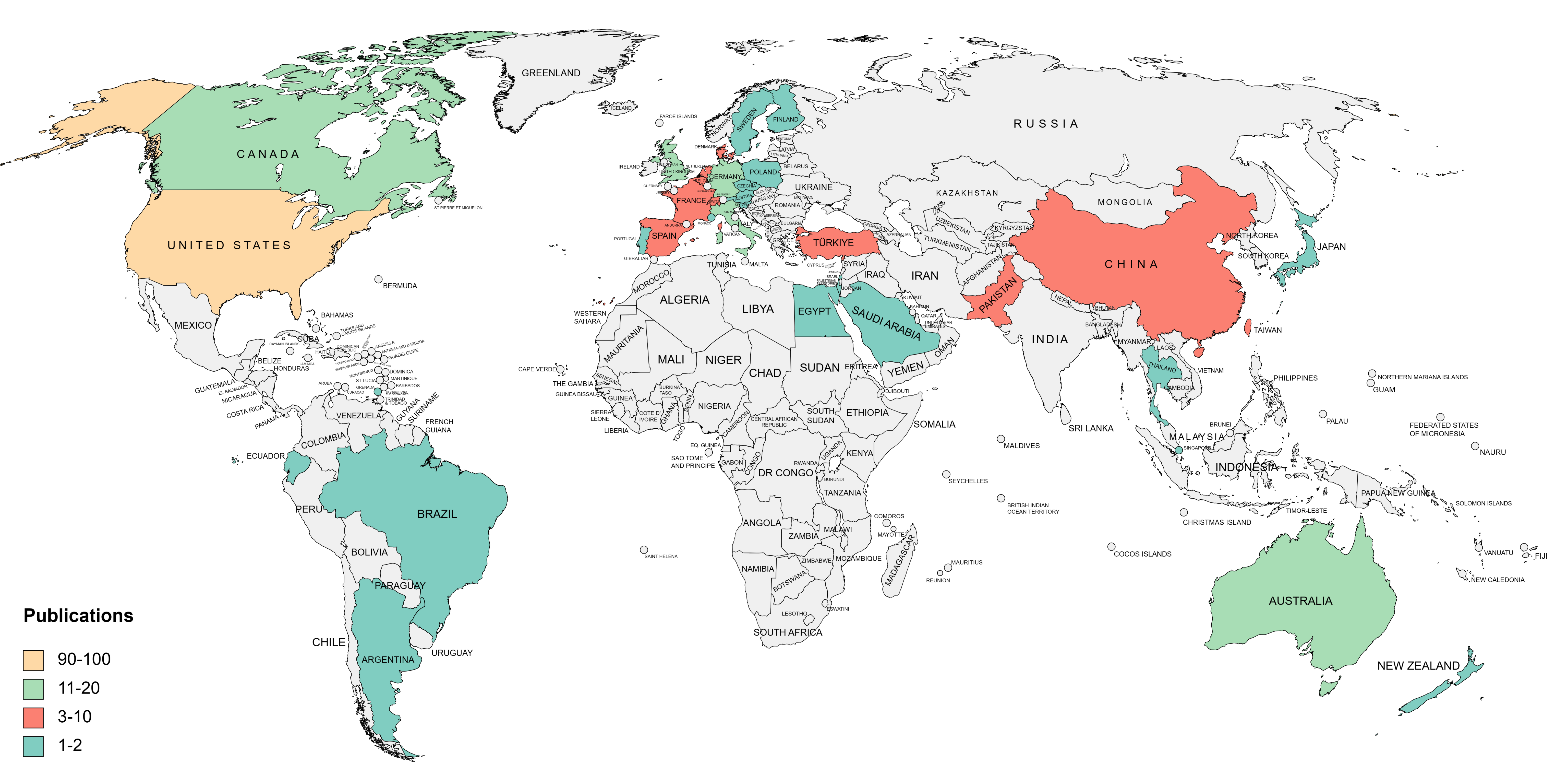


**Appendix S6.8** Cooperation between States, institutions, and authors


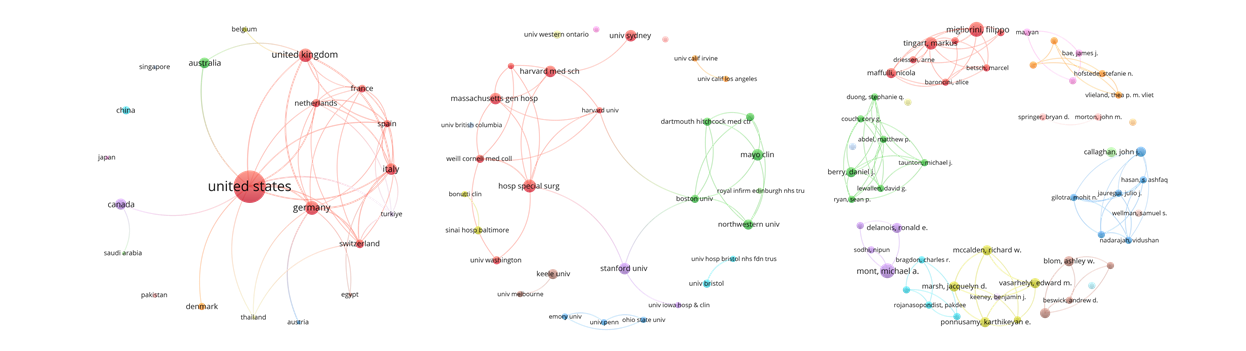


**Appendix S6.9** Keyword relevance analysis.


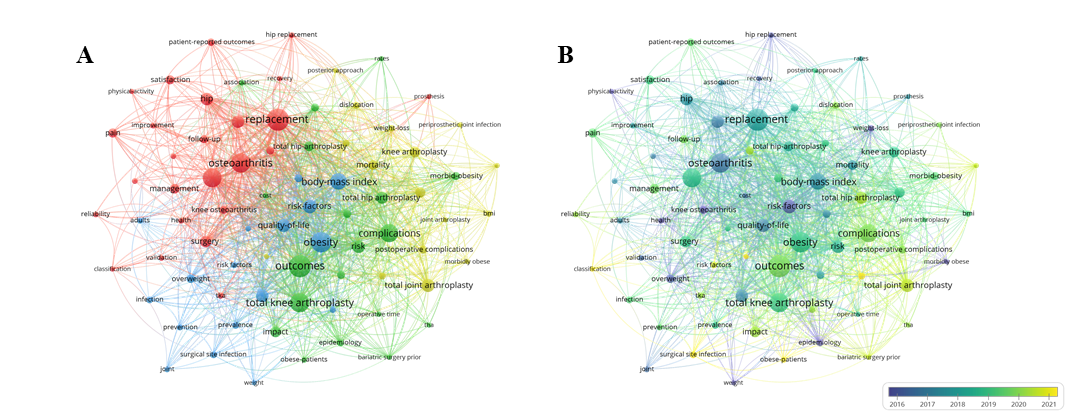


**Appendix S7.** Systemic review of influence of obesity on surgical outcomes of joint replacement.

**Appendix S7.1** PRISMA flow chart of study identification, screening, and selection for the obesity and joint replacement surgical outcomes

**
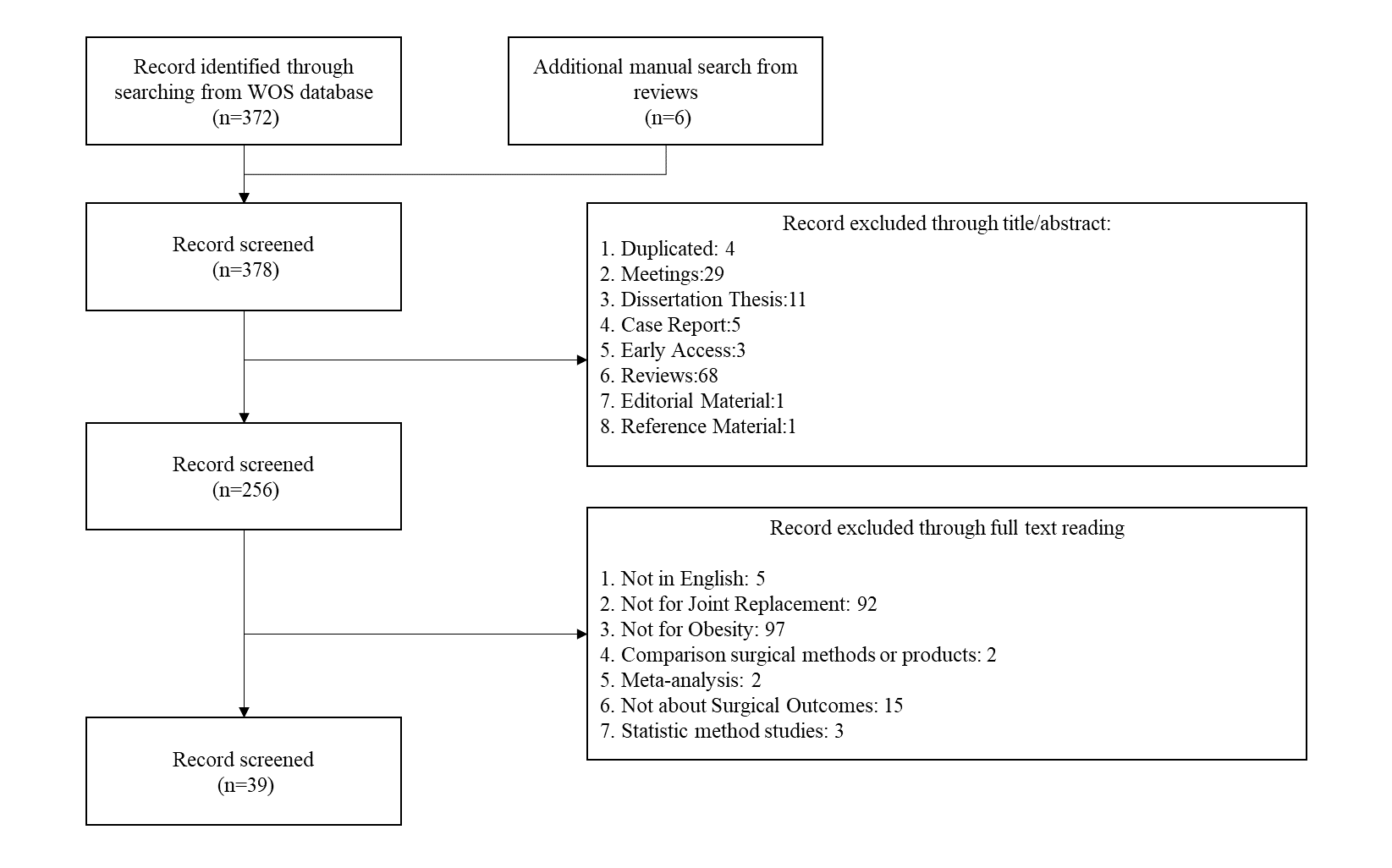
**

**Appendix S7.2** Comprehensive review chart of systemically selected study populations, findings, and recommendations.

| Author | Study Population | Main result and recommendation |
| --- | --- | --- |
| Elcock, K. L. et al.[1] | The study population consisted of 111 patients with severe obesity who underwent total knee arthroplasty (TKA) and had a body mass index (BMI) of ≥40 kg/m^2^. | Favorable knee arthroplasty outcomes in BMI ≥ 40 kg/m^2^ with different tibial baseplates. |
| Abella, M., et al.[2] | This study included 235,061 elective, unilateral total hip arthroplasty (THA) cases from the National Surgical Quality Improvement Project (NSQIP) database to analyze the impact of operative time on obesity-related outcomes. | Operative time is associated with increased risk of readmission, reoperation, and postoperative medical complications in patients with obesity undergoing total hip arthroplasty (THA). |
| Dlott, C.C., et al. [3] | The study population in this study consists of the top 50 orthopedic hospitals in the United States, as ranked by the 2020 US News and World Report's "Best Hospitals for Orthopedics" | Surgeons should engage in shared decision-making with patients regarding risk factors such as BMI, hemoglobin A1c, and smoking, instead of relying solely on strict cutoffs. |
| Bains, S.S., et al. [4] | Patients undergoing primary total hip arthroplasty (THA) from January 2010 to October 2020. The patients were divided into six cohorts: two cohorts without history of bariatric surgery (BMI 20-35 and BMI > 40), two cohorts with previous Roux-en-Y gastric bypass (RYGB) or sleeve gastrectomy (SG), and two cohorts that underwent bariatric surgery either 6-12 months or >12 months prior to THA. | Patients undergoing Roux-en-Y gastric bypass (RYGB) or sleeve gastrectomy (SG) 6-12 months and >1 year prior to total hip arthroplasty (THA) showed similar complication profiles. The recommendation from the study suggests that bariatric patients do not need to wait 1 year before undergoing THA. |
| Rankin, K.A., et al.[5] | Morbidly obese patients who may require total joint arthroplasty (TJA). | The recommendation includes tailored preoperative, intraoperative, and postoperative considerations to ensure safer surgical outcomes for these patients. |
| Abbas, Z., et al.[6] | One hundred and five patients who underwent total knee replacement surgery at the orthopedic department of Shifa International Hospital in Islamabad between June 2021 and December 2021. | Obese patients had a significantly longer duration of surgery compared to non-obese patients. The study recommends that surgeons take into consideration the increased surgical time in obese patients when planning total knee replacement surgeries and allocate sufficient resources accordingly to ensure optimal outcomes. |
| Sax, O.C., et al.[7] | Patients who underwent bariatric surgery (Roux-en-Y Gastric Bypass or sleeve gastrectomy) prior to total knee arthroplasty (TKA) from 2010 to 2020. | Timing (6 months or 1 year prior to TKA) and type of BS shared similar complication profiles, lower than BMI > 40 and higher than BMI 20-35. These findings support a surgeon's decision to proceed with TKA at six months post-BS if indicated. |
| Muthusamy, N., et al.[8] | All patients ≥18 years of age from January 2013 through December 2020 who underwent primary, elective TKA and those who had an annual routine physical examination at our institution within the same period. | Patients who underwent TKA continued to have higher BMI than the general population, which showed a steady increase over time. Physicians need to continue in their efforts to educate patients on weight management and healthy lifestyles to potentially delay the need for a surgical procedure. |
| Kim, B.I., et al.[9] | A retrospective review of 3058 primary TKAs at an academic institution from 2015 to 2019. | Weight gain postoperatively was associated with inferior outcomes. Significant weight loss before surgery led to a "rebound" in weight gain, and independently increased risk for all-cause revision. Therefore, current recommendations for weight loss before TKA in morbidly obese patients should be re-evaluated. |
| Purcell, S., et al.[10] | A retrospective chart review of 355 patients undergoing laparoscopic sleeve gastrectomy from July 2006 to July 2016 at a university hospital was performed | Orthopedic intervention can be delayed and potentially avoided by undergoing LSG. Referral to bariatric surgery should be considered for patients with morbid obesity and severe knee osteoarthritis. |
| Evans, J.T., et al.,[11] | 493,710 TKRs in the National Joint Registry (NJR) for England, Wales, Northern Ireland, and the Isle of Man from 2005 to 2016 | no evidence of increased mortality, and difference in change in Oxford knee score below the minimal detectable change, this large national registry shows no evidence of poorer outcomes in patients with high BMI |
| Zhang, J., et al.[12] | 12,109 unilateral THRs in obese patients. | The choice of surgical approach in obese patients conveys no advantage in overall revision rates in the short-term. Choosing an appropriate size of femoral head may be of greater importance than choice of surgical approach for obese patients in primary THR. |
| Goh, G.S., et al. [13] | Registry data of 192 obese patients and 192 propensity score-matched controls who underwent primary THA at a single institution | Despite a higher revision rate, obese patients undergoing THA may experience a similar level of clinical meaningful improvement and satisfaction as their non-obese counterparts. |
| Shaka, H., et al. [14] | The study population in this study consists of adults with hip osteoarthritis (OA) who underwent hip arthroplasty. The data was obtained from the Nationwide Inpatient Sample (NIS) database for the years 2016 and 2017. | No difference in mortality among obese and non-obese patients who had hip arthroplasty. Obese adults have increased odds of morbidity and perioperative complications. Hence, obese adults likely require better perioperative management to decrease the incidence of complications. |
| Foreman, C.W., et al. [15] | 158 patients with BMI ≥40 kg/m^2^and moderate/severe OA of the hip or knee | A relatively lower BMI indicates a greater chance of retention in care, and ultimately surgery, but does not influence surgeons' recommendations to continue orthopedic management. Patients who persist in seeking treatment, lose significant weight, and exhaust nonoperative alternatives may be suitable for TJA despite a BMI ≥40 kg/m^2^, with an overall complication rate of 4.3%. However, only 9% of patients at 2-year follow-up achieved BMI <40 kg/m^2^and only 20% of surgeries were performed on patients who had achieved this proposed cutoff. |
| Meller, M. M., et al. [16] | 25,852 patients who underwent bariatric surgery followed by total knee arthroplasty (TKA) | (1) Submitting to bariatric surgery is not sufficient to normalize risks. (2) The type of previous bariatric procedure is associated with the type of complications encountered. (3) We were unable to attribute TKA to bariatric failures. (4) Health systems and health care providers should be cautious in withholding care for patients with morbid obesity. |
| Ammann, E. M. et al. [17] | Data from 2013 to 2017 were obtained from the Optum Integrated Claims-Clinical Database, which includes linked and deidentified insurance claims and EHR data for commercially insured and Medicare Advantage health plan members. | Obesity is highly prevalent in many surgical populations, obesity diagnosis codes have high PPVs, but also obesity is generally undercoded in claims data. Quantifying the validity of diagnosis codes for obesity and other important prognostic factors is important for the design and interpretation of studies of surgical outcomes based on administrative data. Further research is needed to determine the extent to which undercoding of BMI and obesity can be addressed using proxies that may be better documented in claims data. |
| Naylor, J. M. et al. [18] | 1757 patients who underwent TKA or THA for osteoarthritis from pre-existing nationally-acquired cohort using 3-year telephone follow-up. | Different mechanisms are likely associated with significant weight gain or loss at 3-years post-surgery. Cogent weight management entails consideration of both outcomes. Many post-surgical factors appear not to be importantly associated with weight change. |
| Sloan, M., et al. [19] | 131,579 patients undergoing primary THA, 218,997 patients undergoing primary TKA, 12,913 patients undergoing revision THA, and 15,286 patients undergoing revision TKA between 2008 and 2016, as documented in the American College of Surgeons National Surgical Quality Improvement Program (ACS-NSQIP) database. | Patient classified as overweight or obese is associated with increased risk of development of PE but not DVT after primary THA or TKA. |
| Mouchti, S., et al. [20] | A population-based, longitudinal cohort study of the National Joint Registry (NJR) for England, Wales, Northern Ireland, and the Isle of Man from April 2003 to December 2015. | Long-term revision rates following total hip replacement were higher among obese patients, we believe that the rates remained acceptable by contemporary standards and were balanced by a lower risk of 90-day mortality. |
| Ponnusamy, K. E, et al. [21] | A state-transition Markov model to compare the cost utility of THA and NM in the 6 BMI groups over a 15-year period. | THA would be cost-effective for all obesity levels. BMI cut-offs for THA may lead to unnecessary loss of healthcare access. |
| Tió, M., et al. [22] | 922 patients undergoing TKA in a single institute | Severely and morbidly obese patients did not show greater blood loss nor higher RBC transfusion needs after primary TKA than non-obese and obese Class I patients. |
| George, J. et al. [23] | 150,934 primary TKAs was queried from the National Surgical Quality Improvement Project database from 2011 to 2015. | Obesity increased the risk of readmission and various complications after TKA, with the risk being dependent on the severity of obesity. A potential BMI goal in weight management for obese patients could be established around 29-30 kg/m^2^, to decrease the risk of most TKA postoperative complications. |
| Roche, M. et al. [24] | A total of 87,607 TKA patients within the study BMI ranges. | The cost of treating obese patients rises as BMI deviates from normal, as does the incidence of revision surgery. Therefore, surgeons must be active in counseling patients on weight optimization as part of preoperative standard of care. |
| Ahmed, W., et al. [25] | The cross-sectional study was conducted at Aga Khan University Hospital and comprised patients undergoing TKA for primary knee osteoarthritis. (142 knees) | Significantly higher infection rate is found in the morbidly obese patients. |
| Frisch, N., et al. [26] | Retrospectively evaluated 2399 patients, 896 of whom underwent THA and 1503 of whom underwent TKA. | Patients with an elevated BMI have decreased rates of blood transfusion following both THA and TKA. This same cohort also loses a significantly decreased percentage of estimated blood volume. No trends were identified for a relationship between BMI and deep venous thrombosis, pulmonary embolism, myocardial infarction, discharge location, length of stay, 30-day readmission rate, and preoperative hemoglobin level. Elevated BMI was significantly associated with increased estimated blood loss in patients undergoing THA and those undergoing TKA. There was a statistically significant trend toward increased deep surgical-site infection in patients undergoing THA. Patients with increased BMI have lower rates of blood transfusion and lose a significantly smaller percentage of estimated blood volume following THA and TKA. |
| Ledford, C. K. et al. [27] | Clinical and functional outcomes were collected prospectively in 215 patients undergoing primary total knee arthroplasty (115 patients) or total hip arthroplasty (100 patients) at a mean time of twenty-four months (range, twelve to forty months). | Higher percent body fat predicted occurrence of any medical or surgical complication. Percent body fat should be considered when predicting clinical and functional outcomes at two years following total joint arthroplasty. Percent body fat may help surgeons to improve risk stratifications, to project patient-reported functional outcomes, and to better educate obese patients about postoperative expectations prior to undergoing elective total joint arthroplasty. |
| Craik, J. D. et al. [28] | Patients undergoing hip and knee arthroplasty (joint replacement surgery) in the UK. The data used for the study was obtained from the 2012-2013 UK National Joint Registry (NJR), which includes patient records from that time. The study specifically focuses on obese patients with a body mass index (BMI) of 30kg/m^2^ or higher. | A high proportion of patients are receiving implants against manufacturer recommendations. However, there are limitations to using BMI for stratifying risk of implant fatigue failure and manufacturers should therefore provide more detailed guidelines on size specific implant load limits to facilitate surgical decisions. |
| Werner, B. C., et al. [29] | A total of 6928 patients from a national insurance database was queried for ORIF or TEA for management of a distal humerus fracture using procedural and diagnostic codes. | Obesity is associated with significantly higher rates of complications after ORIF and TEA for distal humerus fractures than in nonobese patients. |
| Russo, M. W., et al. [30] | 210 consecutive patients who underwent unilateral total hip arthroplasty (THA) through a direct anterior (DA) approach. | The study found that obese patients undergoing total hip arthroplasty (THA) through a direct anterior approach had longer surgical times, increased length of stay, higher complication rates, and greater use of narcotics and assistive devices compared to non-obese patients. |
| Bouchard, M., et al. [31] | 87 patients undergoing total ankle replacement for end-stage ankle arthritis. It included thirty-nine obese patients (body mass index ≥30 kg/m²) and forty-eight non-obese patients (body mass index <30 kg/m²). Ten of the obese patients were morbidly obese (body mass index >40 kg/m²). | Although obese patients had increased disability and worse function preoperatively, total ankle replacement significantly and similarly improved pain and disability scores in both obese and non-obese patients, with no significant difference in the proportion of complications. We therefore maintain that total ankle replacement is a reliable treatment option for patients with end-stage ankle arthritis, including those who are obese. |
| Watts, C. D., et al. [32] | 111 patients who underwent two-stage revision total knee arthroplasty for periprosthetic joint infection. This included thirty-seven morbidly obese patients (body mass index ≥40 kg/m²) and seventy-four matched non-obese patients (body mass index <30 kg/m²). | Morbidly obese patients had a higher risk of revision surgery, reinfection, and reoperation compared to non-obese patients. Despite significant pain relief following surgery in both groups, morbidly obese patients experienced worse pain relief and overall function. The study recommends anticipating increased failure rates and poorer outcomes in morbidly obese patients, although two-stage revision remains the standard treatment for chronic periprosthetic joint infection. |
| Motaghedi, R., et al. [33] | 60 patients undergoing elective primary unilateral total hip arthroplasty (THA). It included three groups: 20 normal-weight, 20 overweight, and 20 obese patients. | Obesity is associated with an enhanced proinflammatory state post-THA, as shown by increased cytokine reactivity. No correlation was found between obesity and increased postoperative pain or analgesic consumption. The study recommends further research to explore the specific impact of obesity and inflammation on surgical outcomes, including pain, in larger studies. |
| Li, X., et al. [34] | 76 patients undergoing primary total shoulder arthroplasty, categorized by body mass index (BMI) into three groups: 26 normal-weight (BMI < 25 kg/m²), 25 overweight (BMI 25-29.9 kg/m²), and 25 obese (BMI ≥30 kg/m²). | Obesity did not negatively impact short-term shoulder function improvement post-arthroplasty. However, overall physical function did not significantly improve in obese and overweight patients, unlike in the normal BMI group. The study recommends recognizing that while shoulder function improves in obese patients’ post-arthroplasty, their overall physical function may not significantly enhance, differing from normal-weight patients. |
| Naziri, Q. et al. [35] | 95 patients (21 men, 74 women) who had undergone primary total knee arthroplasty (TKA), with 101 knees being operated. The patients were super-obese, with a minimum BMI of 50 kg/m², and were compared to a matched group with a BMI of less than 30 kg/m². | Super-obese patients had significantly higher rates of medical and surgical complications and lower functional outcomes compared to the non-obese group. No significant differences in implant survivorship were observed. The study recommends recognizing the increased risks and poorer outcomes in super-obese patients undergoing TKA. |
| Lübbeke, A et al. [36] | 433 patients who underwent 503 hip arthroplasties, categorized by body mass index: normal weight (<25 kg/m²), overweight (25-29.9 kg/m²), and obese (≥30 kg/m²). | No increased risk of osteolysis around a cemented femoral stem in obese patients five and ten years after primary total hip arthroplasty. Surprisingly, the highest prevalence of osteolysis was in normal-weight patients. The study recommends that obesity should not be considered a significant risk factor for osteolysis in patients with cemented femoral stems in hip arthroplasty. |
| Mulhall, K. J. et al. [37] | 291 consecutive patients undergoing revision total knee arthroplasty (TKA), with an average BMI of 32.3 ± 7.7. 57% of these patients were obese (BMI ≥ 30). | Higher BMI and weight negatively impact the longevity of primary TKA and the functional outcomes and quality of life following revision TKA. Obesity and overweight were significant predictors of reduced survivorship and poorer functional outcomes. The study recommends more effective management strategies for obese and overweight patients undergoing TKA to improve outcomes. |
| Jibodh, S. R. et al. [38] | 207 patients who underwent primary total hip arthroplasty, grouped by body mass index (BMI). | Morbidly obese patients (BMI ≥ 40 kg/m²) experienced longer operative times and higher mean intraoperative blood loss, with a trend toward more complications. However, there was no significant difference in functional recovery and hospital use between the groups. The study suggests careful perioperative planning for morbidly obese patients to manage the increased risks, while noting their similar functional recovery and hospital use compared to others. |
| Böstman O. M. [39] | 2673 patients awaiting common orthopedic surgeries: lumbar intervertebral disc herniation removal, total hip replacement, elective knee arthroscopy, or total knee replacement. Patients with inflammatory joint disease were excluded. | Obesity was more prevalent among orthopedic surgery patients than in the general population, particularly in young patients undergoing lumbar disc herniation surgery and women undergoing knee arthroscopy. The study concludes that overweight individuals are significantly over-represented in patients requiring these common orthopedic procedures, suggesting a need for targeted weight management strategies in this patient group. |

**References**

1. Elcock, K.L., et al., *Total knee arthroplasty in patients with severe obesity: outcomes of standard keeled tibial components versus stemmed universal base plates.* Knee Surg Relat Res, 2023. **35**(1): p. 9.

2. Abella, M., et al., *Does Operative Time Modify Obesity-related Outcomes in THA?* Clin Orthop Relat Res, 2023. **481**(10): p. 1917-1925.

3. Dlott, C.C., et al., *Preoperative Risk Management Programs at the Top 50 Orthopaedic Institutions Frequently Enforce Strict Cutoffs for BMI and Hemoglobin A1c Which May Limit Access to Total Joint Arthroplasty and Provide Limited Resources for Smoking Cessation and Dental Care.* Clin Orthop Relat Res, 2023. **481**(1): p. 39-47.

4. Bains, S.S., et al., *Bariatric surgery prior to total hip arthroplasty: does timing or type matter?* Hip Int, 2023. **33**(6): p. 1017-1025.

5. Rankin, K.A., et al., *Operative Techniques to Reduce Hip and Knee Arthroplasty Complications in Morbidly Obese Patients.* Arthroplast Today, 2022. **17**: p. 120-125.

6. Abbas, Z., et al., *Effect of body mass index on duration of total knee replacement surgery: A prospective cross sectional study.* Ann Med Surg (Lond), 2022. **82**: p. 104637.

7. Sax, O.C., et al., *Timing and Type of Bariatric Surgery Preceding Total Knee Arthroplasty Leads to Similar Complications and Outcomes.* J Arthroplasty, 2022. **37**(8s): p. S842-s848.

8. Muthusamy, N., et al., *Trends of Obesity Rates Between Patients Undergoing Primary Total Knee Arthroplasty and the General Population from 2013 to 2020.* J Bone Joint Surg Am, 2022. **104**(6): p. 537-543.

9. Kim, B.I., et al., *Preoperative Weight Loss and Postoperative Weight Gain Independently Increase Risk for Revision After Primary Total Knee Arthroplasty.* J Arthroplasty, 2022. **37**(4): p. 674-682.

10. Purcell, S., et al., *Morbid Obesity and Severe Knee Osteoarthritis: Which Should Be Treated First?* J Gastrointest Surg, 2022. **26**(7): p. 1388-1393.

11. Evans, J.T., et al., *Obesity and revision surgery, mortality, and patient-reported outcomes after primary knee replacement surgery in the National Joint Registry: A UK cohort study.* PLoS Med, 2021. **18**(7): p. e1003704.

12. Zhang, J., et al., *Does surgical approach affect early outcomes following primary total hip replacement in obese patients?* Hip Int, 2021. **31**(3): p. 304-310.

13. Goh, G.S., et al., *Does obesity lead to lower rates of clinically meaningful improvement or satisfaction after total hip arthroplasty? A propensity score-matched study.* Hip Int, 2022. **32**(5): p. 610-619.

14. Shaka, H. and P.E. Ojemolon, *Impact of Obesity on Outcomes of Patients With Hip Osteoarthritis Who Underwent Hip Arthroplasty.* Cureus, 2020. **12**(10): p. e10876.

15. Foreman, C.W., et al., *Total Joint Arthroplasty in the Morbidly Obese: How Body Mass Index ≥40 Influences Patient Retention, Treatment Decisions, and Treatment Outcomes.* J Arthroplasty, 2020. **35**(1): p. 39-44.

16. Meller, M.M., et al., *Does Bariatric Surgery Normalize Risks After Total Knee Arthroplasty? Administrative Medicare Data.* J Am Acad Orthop Surg Glob Res Rev, 2019. **3**(12).

17. Ammann, E.M., et al., *Assessment of obesity prevalence and validity of obesity diagnoses coded in claims data for selected surgical populations: A retrospective, observational study.* Medicine (Baltimore), 2019. **98**(29): p. e16438.

18. Naylor, J.M., et al., *Patient factors associated with weight gain and weight loss after knee or hip arthroplasty.* Obes Res Clin Pract, 2019. **13**(4): p. 371-377.

19. Sloan, M., N. Sheth, and G.C. Lee, *Is Obesity Associated With Increased Risk of Deep Vein Thrombosis or Pulmonary Embolism After Hip and Knee Arthroplasty? A Large Database Study.* Clin Orthop Relat Res, 2019. **477**(3): p. 523-532.

20. Mouchti, S., et al., *The Association of Body Mass Index with Risk of Long-Term Revision and 90-Day Mortality Following Primary Total Hip Replacement: Findings from the National Joint Registry for England, Wales, Northern Ireland and the Isle of Man.* J Bone Joint Surg Am, 2018. **100**(24): p. 2140-2152.

21. Ponnusamy, K.E., et al., *Cost-Effectiveness of Total Hip Arthroplasty Versus Nonoperative Management in Normal, Overweight, Obese, Severely Obese, Morbidly Obese, and Super Obese Patients: A Markov Model.* J Arthroplasty, 2018. **33**(12): p. 3629-3636.

22. Tió, M., et al., *Severe and morbid obesity and transfusional risk in total knee arthroplasty: An observational study.* Knee, 2018. **25**(5): p. 923-931.

23. George, J., et al., *Association Between Body Mass Index and Thirty-Day Complications After Total Knee Arthroplasty.* J Arthroplasty, 2018. **33**(3): p. 865-871.

24. Roche, M., et al., *Effect of Obesity on Total Knee Arthroplasty Costs and Revision Rate.* J Knee Surg, 2018. **31**(1): p. 38-42.

25. Ahmed, W., et al., *Does obesity affects early infection after total knee arthroplasty. A comparison of obese vs non obese patients.* J Pak Med Assoc, 2016. **66(Suppl 3)**(10): p. S96-s98.

26. Frisch, N., et al., *Effect of Body Mass Index on Blood Transfusion in Total Hip and Knee Arthroplasty.* Orthopedics, 2016. **39**(5): p. e844-9.

27. Ledford, C.K., et al., *Percent Body Fat Is More Predictive of Function After Total Joint Arthroplasty Than Body Mass Index.* J Bone Joint Surg Am, 2016. **98**(10): p. 849-57.

28. Craik, J.D., M.D. Bircher, and M. Rickman, *Hip and knee arthroplasty implants contraindicated in obesity.* Ann R Coll Surg Engl, 2016. **98**(5): p. 295-9.

29. Werner, B.C., et al., *Obesity is associated with increased postoperative complications after operative management of distal humerus fractures.* J Shoulder Elbow Surg, 2015. **24**(10): p. 1602-6.

30. Russo, M.W., et al., *Increased Complications in Obese Patients Undergoing Direct Anterior Total Hip Arthroplasty.* J Arthroplasty, 2015. **30**(8): p. 1384-7.

31. Bouchard, M., et al., *The impact of obesity on the outcome of total ankle replacement.* J Bone Joint Surg Am, 2015. **97**(11): p. 904-10.

32. Watts, C.D., et al., *Morbid obesity: a significant risk factor for failure of two-stage revision total knee arthroplasty for infection.* J Bone Joint Surg Am, 2014. **96**(18): p. e154.

33. Motaghedi, R., et al., *Association of obesity with inflammation and pain after total hip arthroplasty.* Clin Orthop Relat Res, 2014. **472**(5): p. 1442-8.

34. Li, X., et al., *Functional outcomes after total shoulder arthroplasty in obese patients.* J Bone Joint Surg Am, 2013. **95**(21): p. e160.

35. Naziri, Q., et al., *Bariatric orthopaedics: total knee arthroplasty in super-obese patients (BMI > 50 kg/m2). Survivorship and complications.* Clin Orthop Relat Res, 2013. **471**(11): p. 3523-30.

36. Lübbeke, A., et al., *Influence of obesity on femoral osteolysis five and ten years following total hip arthroplasty.* J Bone Joint Surg Am, 2010. **92**(10): p. 1964-72.

37. Mulhall, K.J., et al., *Adverse effects of increased body mass index and weight on survivorship of total knee arthroplasty and subsequent outcomes of revision TKA.* J Knee Surg, 2007. **20**(3): p. 199-204.

38. Jibodh, S.R., I. Gurkan, and J.F. Wenz, *In-hospital outcome and resource use in hip arthroplasty: influence of body mass.* Orthopedics, 2004. **27**(6): p. 594-601.

39. Böstman, O.M., *Prevalence of obesity among patients admitted for elective orthopaedic surgery.* Int J Obes Relat Metab Disord, 1994. **18**(10): p. 709-13.
